# Green synthesis of quinazoline derivatives using a novel recyclable nano-catalyst of magnetic modified graphene oxide supported with copper

**DOI:** 10.1038/s41598-023-48120-6

**Published:** 2023-11-28

**Authors:** Sarieh Momeni, Ramin Ghorbani‑Vaghei

**Affiliations:** Department of Organic Chemistry, Faculty of Chemistry, Bu-Ali Sina University, Hamedan, Iran

**Keywords:** Chemistry, Materials science, Nanoscience and technology

## Abstract

A new magnetic nano-catalyst system based on graphene oxide was designed and manufactured (GO@Fe_3_O_4_@3-chloropropyltrimethoxysilane@(Z)-*N'*-(2-hydroxybenzylidene)-4-(pyridin-4-yl)benzohydrazide@Cu(II)), and it was checked and confirmed by various analyzes such as FTIR, XRD, EDX, MAPPING, TGA/DSC, VSM and FESEM. This nano-catalyst was used in the three-component one-pot synthesis of quinazoline derivatives. The products were obtained using this efficient catalyst with high efficiency in short time and solvent-free conditions. Easy separation and acceptable recyclability are other advantages of this new nano-catalyst. Also, the catalyst can be recycled 4 times without a significant change in its efficiency.

## Introduction

Carbon nanomaterials are used in the structure of various chemical compounds such as graphite, fullerenes, carbon nanotubes and graphene, that is why it has attracted the attention of researchers^1–3^ . The structure of graphene is in the form of two-dimensional layers that are not completely flat, rather flexible and have a wavy, curved and folded surface. If these bends are large, are related to the graphene synthesis method, and if they are small, are inherent characteristics of graphene^4,5^. Properties such as high current density, chemical inertness, high thermal conductivity, the highest electron transfer rate, high thermal conductivity, and unique mechanical and electrochemical^6–8^ of graphene oxide, make it valuable for various applications such as catalysts, batteries, supercapacitors, sensors, and biotechnology^9–11^. Brody, Staudenmaier and Hammer methods are used for graphene synthesis. The most famous method for the synthesis of graphene is Hamer's method, in which graphite is oxidized by oxidizing compounds such as potassium permanganate and hydrogen peroxide in the presence of sulfuric acid and created graphene oxide^12,13^. Due to the presence of functional groups such as carboxyl, hydroxyl, carbonyl, and epoxide groups, it easily disperses in water and polar solvents, but does not disperse in non-polar solvent, that by functionalizing it with organic compounds, it dispersed in non-polar solvents and created a stable suspension^14^. Iron nanoparticles are used in an attractive field of science and industry due to many applications such as solar cells, semiconductors, catalysts, targeted drug delivery and tissue engineering^15–17^. Due to its high surface-to-volume ratio, highly active surface, ability to combine with various functional groups, non-toxicity, low cost, high thermal and chemical stability, and easy separation ability, it is of great interest as a catalyst or support for catalytic processes in the synthesis of heterocyclic compounds^18–20^. They are easily separated from the reaction mixture with an external magnet, easily recycled, and the purification process is easy, due to the simplicity of the recovery operation of magnetic catalysts which is an eco-friendly and green process, have recently received much attention^21–23^. It is difficult to separate graphene from the reaction medium by centrifugation and filtration, but if it is combined with iron, it can be easily separated and reused, which has received a lot of attention recently^24–26^. Various synthetic methods for magnetized graphene have been proposed, including adding FeCl_3_ to a hot mixture of NaOH and diethylene glycol, hydrothermal, ion exchange and subsequent calcination, chemical precipitation, binding Fe_3_O_4_ NPs to GO through covalent bonding, and microwave irradiation. Multicomponent reactions with high atom saving, high selectivity, fast and economical are one of the most flexible ways to synthesize heterocyclic compounds with high medicinal and biological capabilities^27–29^. In this method, a wide range of products can be synthesized by minimizing waste, cost, time and without separating intermediates. Quinazoline compounds are heterocyclic compounds containing nitrogen atom, which have attracted the attention of many researchers due to their many medicinal properties. Among their medicinal properties can mention anti-cancer^30^, anti-malarial^31^, anti-bacterial^32^, anti-inflammatory^33^, anti-asthma^34^, Epidermal growth factor receptor (EGFR) inhibitors^35^, anti-blood pressure, etc. For this reason, new and better methods for the synthesis of quinazolines have attracted the attention of researchers. Several methods have been reported for the synthesis of quinazolines, some of which can be mentioned, such as the photochemical method, microwave radiation^36^, copper^37^, TBBDA^38^, CoFe_2_O_4_@B_2_O_3_-SiO_2_^39^, butylmethylimidazolium tetrachloroferrate (bmim[FeCl4])^40^, benzylic amines^41^, acetic acid^42^, Fe_3_O_4_@SiO_2_@TiO_2_-OSO_3_H^43^, ruthenium catalytic system ([Ru]/L)^44^, MnO_2_^45^, NaClO^46^, and 1-methylimidazolium trifluoroacetate ([Hmim]TFA)^28^. The mentioned methods have disadvantages such as low yield of products, high reaction time, expensive, toxic, or non-recyclable catalysts.

Here we report a new synthetic method for the synthesis of quinazoline derivatives, by combining magnetic nanoparticles with graphene oxide, we turned it into an efficient, stable, selective and reusable catalytic base, and by stabilizing the metal complex including ligand and copper acetate metal on it (GO@Fe_3_O_4_@3-chloropropyltrimethoxysilane@(Z)-*N'*-(2-hydroxybenzylidene)-4-(pyridin-4-yl)benzohydrazide@Cu(II) NPs), this recyclable heterogeneous catalyst was used for the synthesis of various derivatives of quinazolines through a one-pot three-component reaction of ammonium acetate, 2-amino-5-chlorobenzophenone and various aldehydes under green conditions (Scheme 1). This method had several advantages, including the reusability of the catalyst, high selectivity, easy purification, easily accessible starting materials, soft reaction conditions, high yield of products, and short reaction time.

## Experimental

### Materials and equipment

All solvents and chemicals were purchased from Merck and used without further purification. ^1^H NMR and ^13^C NMR spectra were recorded using a Bruker BioSpin GmbH 500 MHz FT NMR spectrometer. Melting points were obtained using a BUCHI 510 apparatus in open tubes. Fourier transform infrared (FT-IR) spectra were obtained using KBr pellets in the range of 400–400 cm using Perkin Elmer GX FT-IR instruments. Field emission scanning electron microscope (FESEM) images were recorded using TESCAN MIRA3 FE-SEM. Energy dispersive X-ray analysis (EDX) was recorded by an EDAX-EDS device. Thermogravimetric analysis (TGA) and differential scanning calorimetry (DSC) were performed by a TGA-DTA device with a heating rate of 10 °C/min in an N_2_ gas atmosphere in the temperature range of 40–550 °C. X-ray diffraction (XRD) patterns were measured by a Philips PW1730 device in the range of 10 to 90 degrees (2θ). VSM was recorded using a Lakeshore 740 device. Evaluation of reaction process was done by thin-layer chromatography (TLC).

### General procedure for preparation of graphene oxide (GO)

Graphene oxide was prepared from natural graphite according to the modified Hammers method. First, a 250 mL balloon was placed in an ice bath and 50 mL of 98% sulfuric acid was added to it with 0.5 g of graphite being stirred. After 15-min, 2 g of potassium permanganate was added to it slowly and gradually over 2 h. After three hours, 150 mL of distilled water was slowly and cautiously added to it, and after half an hour, 10 mL of 30% hydrogen peroxide were added slowly. And for washing, 5% hydrochloric acid and distilled water are used and washed several times with a centrifuge, then it is placed in an oven for 24 h at a temperature of 60 °C^13^.

### General procedure for preparation of GO/Fe_3_O_4_

40 mg of graphene oxide was poured into 40 mL of distilled water and placed in a Sonicate device for 30-min. Then 50 mL of solution containing 0.21 mmol of FeCl_2_.4H_2_O and 0.4 mmol of FeCl_3_.6H_2_O was added to it and the mixture was heated to 85 C and then 30% ammonia solution was added until the pH of the solution reached 10. And in the following, the resulting mixture was stirred for 45-min, then it was separated with a centrifuge and washed several times with distilled water, and placed in an oven at a temperature of 60 °C for 24 h to dry^47^.

### General procedure for preparation of GO/Fe_3_O_4_@TRMS (3-chloropropyltrimethoxysilane)

GO/Fe_3_O_4_@TRMS was prepared by two methods, in the first method 200 mg of GO/Fe_3_O_4_ was poured into a mixture of water and ethanol in a ratio (4:1) and placed in an ultrasonic device to disperse for 30-min. Then 23 mmol of 3-chloropropyltrimethoxysilane was added to it and the resulting mixture was stirred for 24 h at temperature of 40 °C. After the completion of the reaction, the magnetic nanoplates were separated with a super magnet, and washed three times with a mixture of water and ethanol, and placed in an oven with a temperature of 60 °C for 24 h to dry^14^. In the second method, 200 mg of GO/Fe_3_O_4_ was poured into 30 mL of toluene solvent and placed in an ultrasonic device to disperse for 30-min. Then 15 mmol of 3-chloropropyltrimethoxysilane was added to it and the obtained mixture was stirred for 24 h at reflux temperature. After the completion of the reaction, the magnetic nanosheets were separated with a super magnet and washed three times with toluene and two times with ethanol and placed in an oven at 60 °C for 24 h to dry.

### Procedure for synthesis of (Z)-*N'*-(2-hydroxybenzylidene)-4-(pyridin-4-yl)benzohydrazide (HBPB) ligand

10 mmol of 4-(pyridin-4-yl)benzohydrazide was dissolved in 20 mL of ethanol, and in a separate container, 2 hydroxybenzaldehyde dissolved in ethanol was added little by little (within half an hour) to the 4-(pyridin-4-yl)benzohydrazide container, and then the reaction mixture was stirred at reflux temperature for 24 h. After finishing the reaction, the resulting precipitate is separated by centrifuge, washed several times with ethanol, and was placed in a 60 °C oven for 24 h to dry.

### Procedure for synthesis of GO/Fe_3_O_4_@TRMS@HBPB

A 100 mL flask containing 0.5 g of graphene coated with 3-chloropropyltrimethoxysilane, and 40 mL of toluene was placed in an ultrasonic device for 10-min. Then 0.3 g of the HBPB ligand was added to the mixture and stirred under reflux conditions for 24 h. After the end of the reaction, the nanoplates were collected with a super magnet, washed several times with ethanol, it was placed in a 60 °C oven for 24 h to dry.

### Procedure for synthesis of GO/Fe_3_O_4_@TRMS@HBPB@Cu

**Figure 1:**
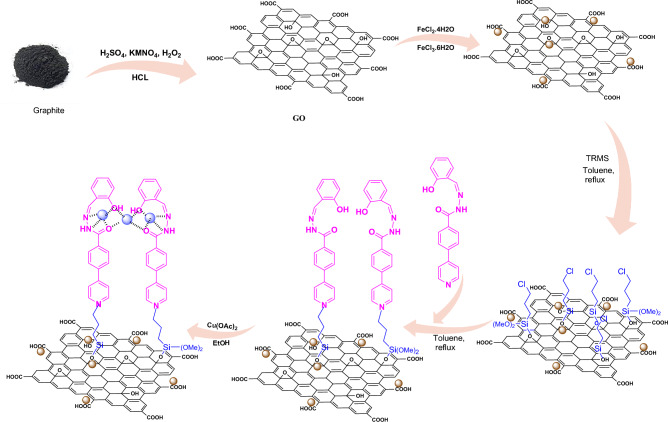
The synthetic route of GO@Fe_3_O_4_@TRMS@HBPB@Cu(II) NPs.

0.5 g of graphene functionalized with the HBPB ligand (GO/Fe_3_O_4_@TRMS@HBPB) and 350 mg of copper acetate were added to 40 mL of ethanol solvent, then the mixture was stirred under reflux conditions for 24 h, and after finishing the reaction, the catalyst was separated with a super magnet and washed several times with ethanol and placed in an oven at 60 °C for 24 h to dry. The catalyst synthesis method is shown in Fig. 1.

### General procedure for one-pot three-component synthesis of quinazoline derivatives

A mixture of 5-chloro-2-aminobenzophenone (1.0 mmol), ammonium acetate (2.5 mmol) and aromatic aldehydes (1 mmol) and the GO@Fe_3_O_4_@TRMS@HBPB@Cu(II) NPs (0.05 g) was stirred at a temperature of 45 °C in an oil bath for a certain period of time, and the reaction was followed by TLC [acetone/n-hexane (2:9)]. After the reaction was completed, the mixture was cooled to room temperature, chloroform (2 ml) was added to the reaction vessel and stirred for 2 min, then the GO@Fe_3_O_4_@TRMS@HBPB@Cu nano-catalyst was easily separated from the reaction solution by a super magnet. After evaporation of the solvent, a pure compound was obtained by recrystallization from ethanol.

## Results and discussion

**Figure 2:**
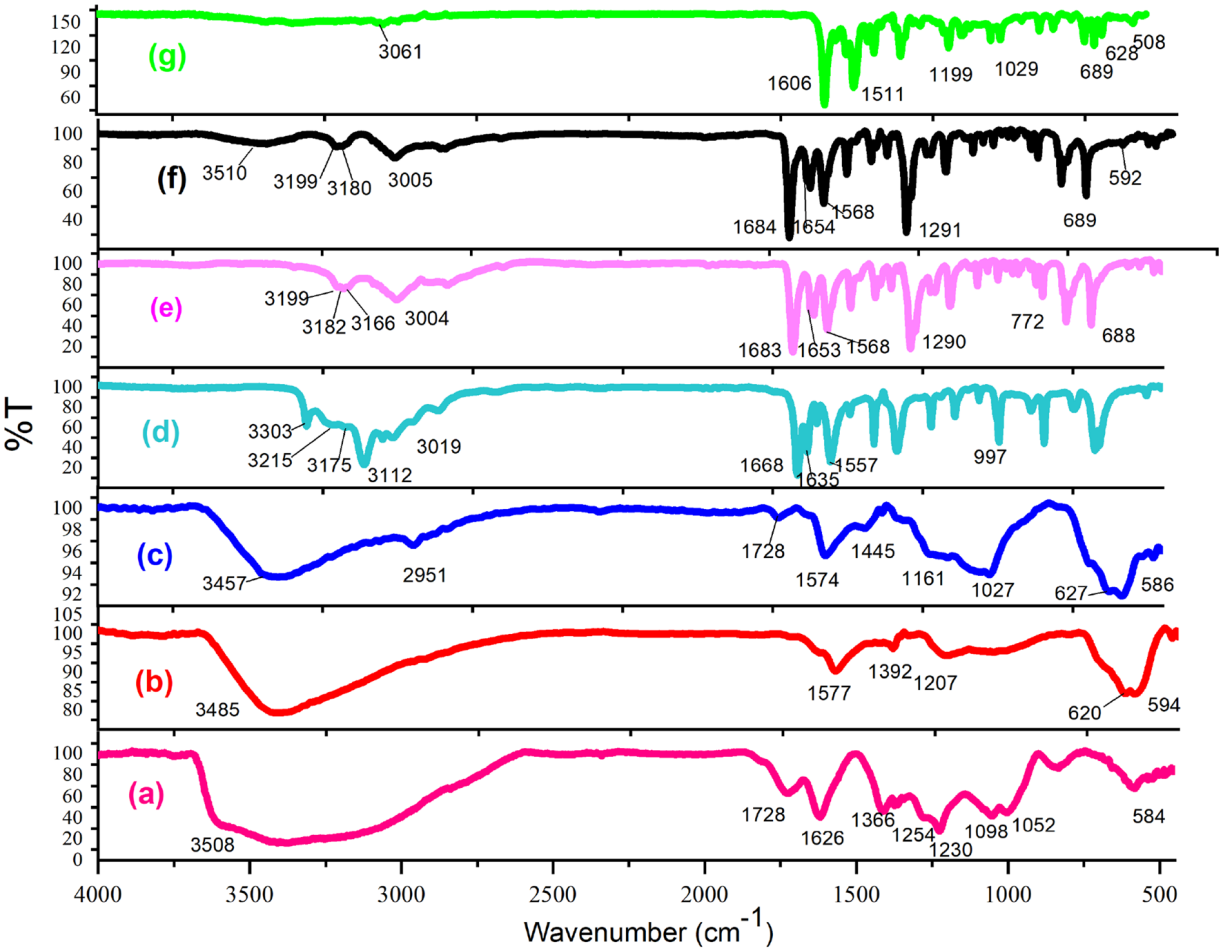
FT-IR spectra of (**a**) GO, (**b**) GO@Fe_3_O_4_, (**c**) GO@Fe_3_O_4_@TRMS, (**d**) 4-amino-(pyridin-4-yl)benzohydrazide, (**e**) HBPB, (**f**) GO@Fe_3_O_4_@TRMS@HBPB, and (**g**) GO@Fe_3_O_4_@TRMS@HBPB@Cu.

**Figure 3:**
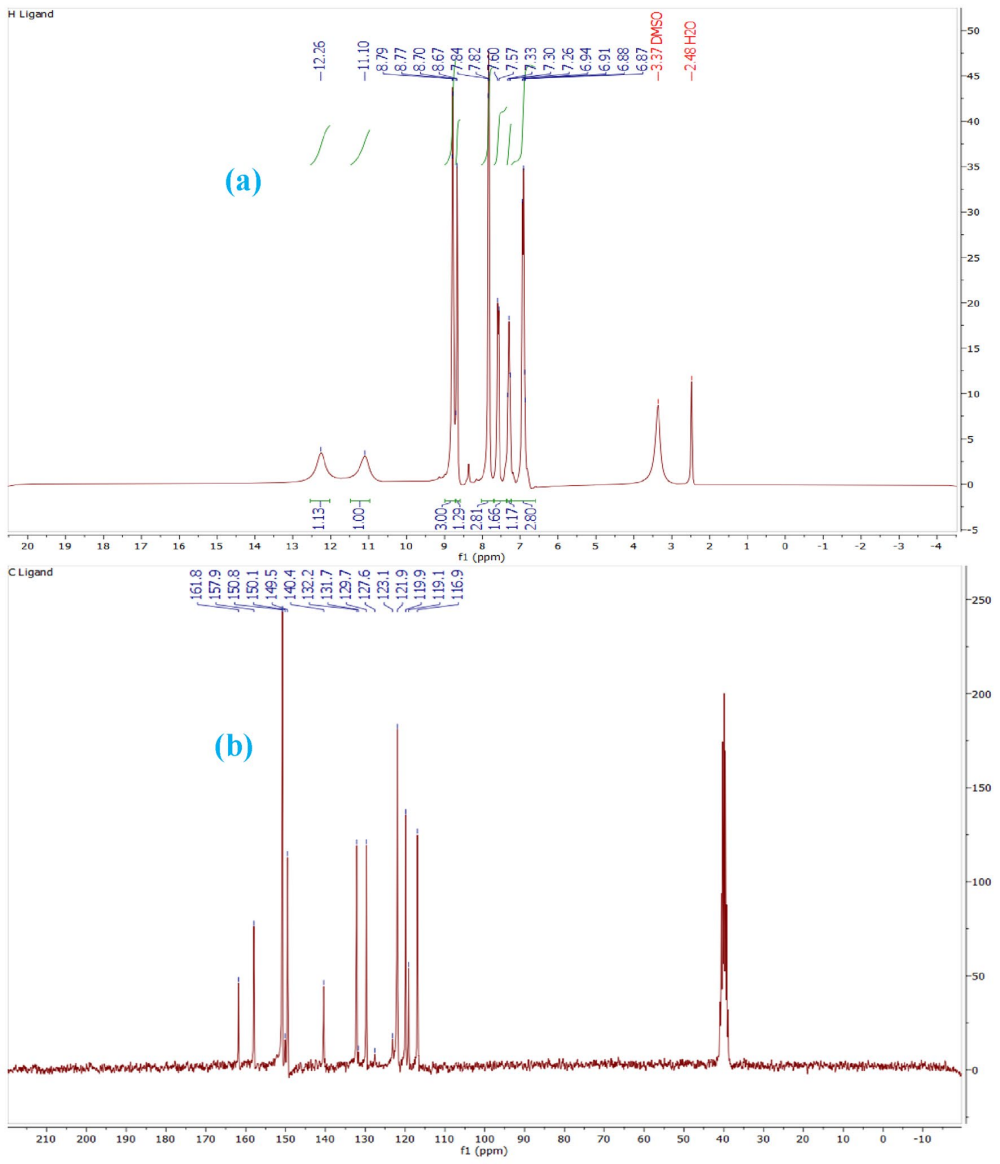
The ^1^H NMR (250 MHz) (**a**) and ^13^CNMR (63 MHz) (**b**) spectrum of the HBPB ligand.

Graphene is obtained from graphite in the presence of permanganate, sulfuric acid and hydrogen peroxide, which contains different functional groups such as epoxy, acid, hydroxyl and ketone. In the next step, magnetized graphene nanosheets GO@Fe_3_O_4_ were created by adding iron to GO, and then functionalized its surface with 3-chloropropyltrimethoxysilane, which reacts more easily with the ligand. Then by placing the synthesized ligand of HBPB, the GO@Fe_3_O_4_@TRMS@HBPB is created and in the last step, the catalyst was synthesized by placing copper acetate on the GO@Fe3O4@TRMS@HBPB. FTIR, FE-SEM, XRD, EDX, MAPPING, DSC and TGA analyses were used to confirm the structure of the catalyst. Figure 2 shows the Fourier transform infrared (FT-IR) spectrum of (a) GO, (b) GO@Fe_3_O_4_, (c) GO@Fe_3_O_4_@TRMS, (d) 4-(pyridin-4-yl)benzohydrazide, (e) ligand of (Z)-*N'*-(2-hydroxybenzylidene)-4-(pyridin-4-yl)benzohydrazide, (f) GO@Fe_3_O_4_@TRMS@HBPB, and (g) GO@Fe_3_O_4_@TRMS@HBPB@Cu. As can be seen in the Fig. 2, in part a, which is related to graphene, there are broad peaks in the region of 3400 cm^−1^, which are related to the stretching vibration of hydroxyl groups on the surface of graphene, and peak 1254 cm^−1^ is the bending vibration of hydroxyl group. The broad peak starting at 2500 cm^−1^ is related the hydroxyl groups of carboxylic acids of graphene. Peak 1728 cm^−1^ is related to the carbonyl stretching vibration of the acid group. The 1366 cm^−1^ peak corresponds to the bending vibration of the carboxyl group, and the 1098 cm^−1^ peak corresponds to the c-o group. The peak of the region of 1626 cm^−1^ is the vibration of the aromatic carbons of the graphitic skeleton, which is not oxidized and is the basis of graphene. The spectrum of part (b), which is related to GO@Fe_3_O_4_ hybrid nanosheets, is different from the spectrum of the previous stage, which completely reduces the intensity of the peaks related to hydroxyl and acidic groups 3400–3500 cm^−1^ and 1728 cm^−1^. The peak related to Fe–O has appeared at 594 cm^−1^ and 620 cm^-1^, which confirms the presence of iron nanoparticles. The interaction between the hydroxyl and carbonyl groups of graphene with iron nanoparticles is proven by the appearance of two absorption bands at 1445 cm^−1^ and 1392 cm^−1^. In part (c), which is related to the chlorination of magnetized graphene oxide, the peak appeared at 2951 cm^-1^ region is related to the aliphatic CH stretching vibrations of 3-chloropropyltrimethoxysilane, which is placed on the magnetic graphene nanosheets. Part (d) is related to 4-amino(pyridin-4-yl)benzohydrazide, by comparing with part (e), which is related to the synthesized HBPB ligand, it is clear that peak appeared at 1683 cm^−1^ is related to the stretching vibrations of the cyanide group formed by the reaction between 2-hydroxybenzaldehyde with 4-amino-(pyridin-4-yl)benzohydrazide, and the stretching vibration of the carbonyl group has shifted from 1668 cm^-1^ to 1653 cm^-1^, and the number of peaks related to NH have decreased, which is related to the formation of the HBPB ligand, the reduction of the stretching vibrations of the amino and amide bonds is due to the reaction of amine with 2-hydroxybenzaldehyde. Part (f) is related to placing the HBPB ligand on the magnetized graphene which was functionalized with 3-chloropropyltrimethoxysilane, it is clear that the spectrum shows a noticeable change, the peaks corresponding to the stretching vibrations of the NH group appear at 3200 cm^-1^ and their bending vibrations at 1100 cm^-1^, the CH stretching vibrations of the benzene ring of ligand appeared at 3005 cm^-1^, and the peak of the hydroxyl group appeared at 3400 cm^-1^, which is related to graphene oxide. In part (g), which is related to the last stage of catalyst manufacturing, where copper acetate is fixed on the composite, the stretching vibration of copper has appeared at 600 cm^-1^ region and the peaks related to NH have decreased significantly, which shows the copper is fixed on nitrogen. Figure 3 shows the ^1^HNMR and ^13^CNMR spectra of the (Z)-*N'*-(2-hydroxybenzylidene)-4-(pyridin-4-yl)benzohydrazide (HBPB) ligand. The part (a) is related to ^1^HNMR of HBPB, the peak appeared at δ 12.26 corresponding to NH, and peak 11 is related to OH group of the ligand, and the peaks of 12 protons related to benzene and pyridine rings appeared at regions of 6.87 to 8.87, while the proton peak of the cyanide double bond appeared at 8.77. In the ^13^CNMR spectrum (part b), the peak 161.8 is related to the carbonyl group and the peak appeared at 157.9 is related to the carbon attached to the hydroxyl group, and the rest of the carbons from 116 to 150 are related to the carbon of rings and double bond.

**Figure 4:**
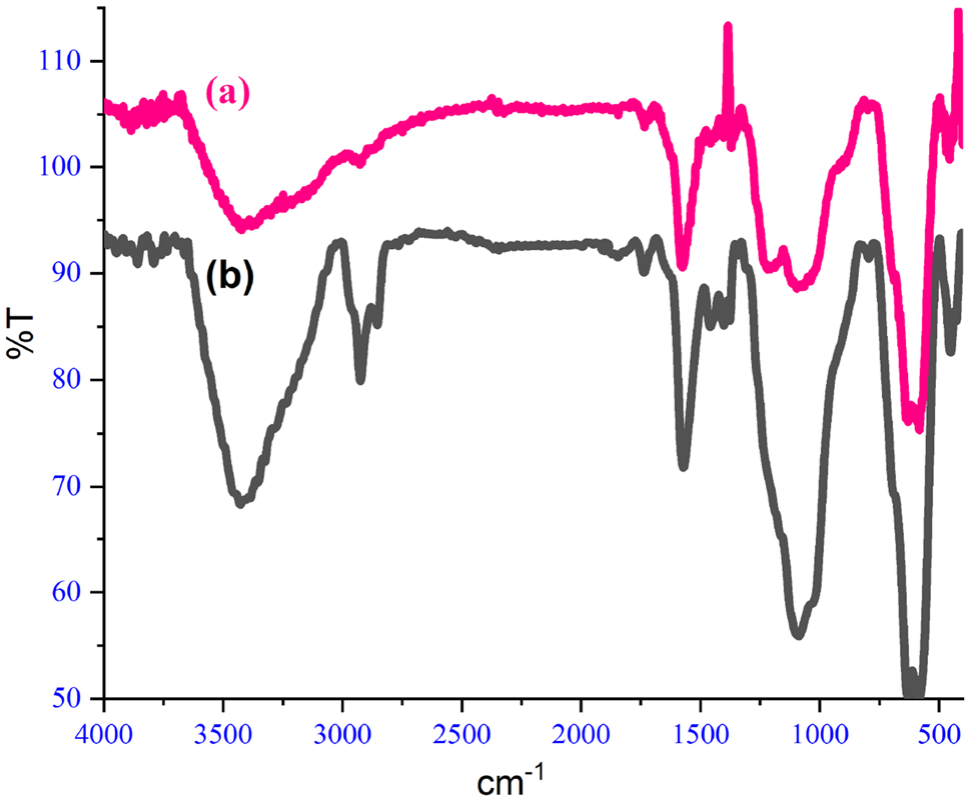
FTIR spectra of (**a**) GO@Fe_3_O_4_@TRMS synthesized in ethanol-water and (**b**) GO@Fe_3_O_4_@TRMS synthesized in toluene solvent.

**Figure 5:**
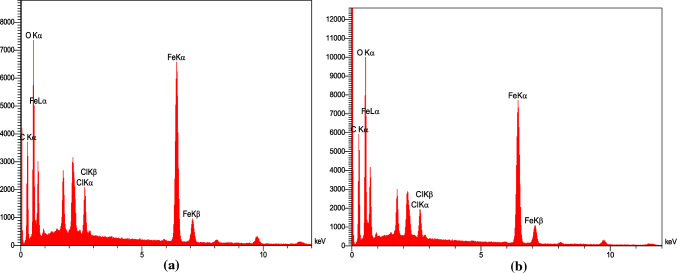
EDX spectra of (**a**) GO@Fe_3_O_4_@TRMS synthesized in ethanol-water solvent and (**b**) GO@Fe_3_O_4_@TRMS synthesized in toluene solvent.

Figure 4 shows the FTIR spectrum of GO@Fe_3_O_4_@TRMS, which is synthesized by two different methods. Part (a) is related to the first method, that water/ethanol solvent was used to place 3-chloropropyltrimethoxysilane on the surface of magnetized graphene oxide, and part (b) is related to the second method, in which toluene solvent was used instead of water/ethanol solvent. It is clear from the figure that the peaks of the two spectra are almost match and the two methods are confirmed. The second method is reported for the first time in this article. Figure 5 is related to EDX images of GO@Fe_3_O_4_@TRMS (image (a) related to the first method and image (b) related to the second method) which are completely similar and confirm the presence of chlorine (Cl). The EDX and FTIR spectra of the two methods show the results of these two methods are not significantly different.

**Figure 6:**
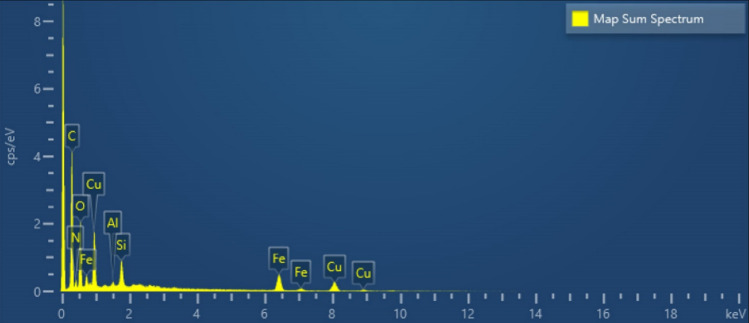
EDX spectra of GO@Fe_3_O_4_@TRMS@HBPB@Cu.

**Figure 7:**
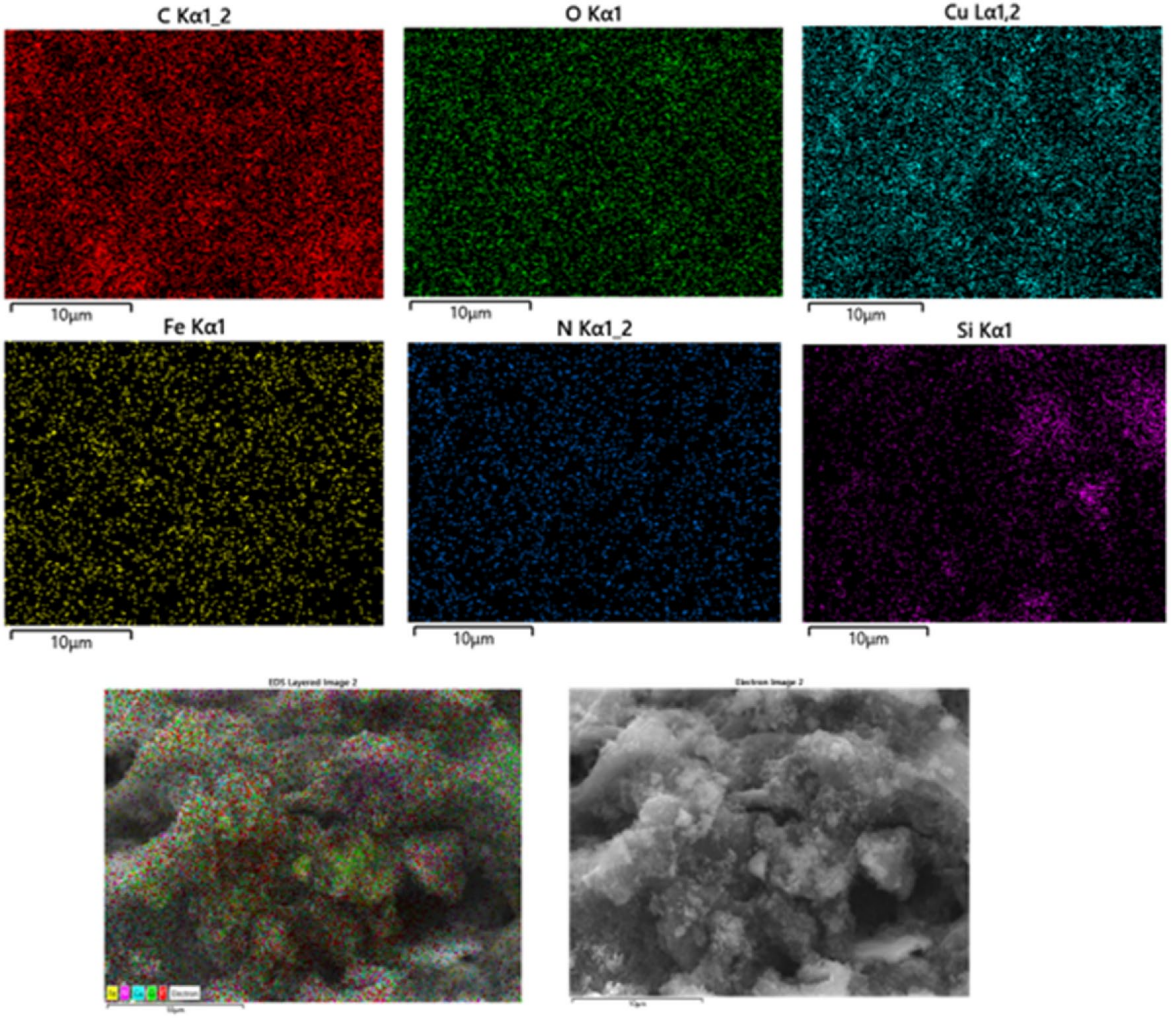
Elemental mapping of C (red); O (green); N (blue); Fe (yellow); Cu (turquoise blue), and Si(purple) atoms for GO@Fe_3_O_4_@TRMS@HBPB@Cu.

According to EDX analysis of the catalyst composition, the peaks related to oxygen, carbon, iron, nitrogen, silicon and copper elements show that the ligand and copper acetate are well placed on the magnetized graphene surface, which is proof of the formation of the corresponding catalyst (Fig. 6). Also, the mapping analysis of the catalyst is shown in Fig. 7. The results of this analysis prove the presence of all expected elements in the structure of this graphene catalyst, including oxygen, carbon, iron, nitrogen, silicon and copper. Table 1 shows the weight and atomic percentages of catalyst GO@Fe_3_O_4_@TRMS@HBPB@Cu The weight and atomic percentage of copper placed on the surface of the catalyst are 8.04 and 1.86, respectively, and the weight and atomic percentage of iron metal placed on the surface of graphene oxide are 4.83 and 1.27, respectively, which are sufficient and appropriate amounts in the catalyst.

**Table 1:** Quantitative amounts of present elements in the GO@Fe_3_O_4_@TRMS@HBPB@Cu catalyst obtained from EDX analysis.

Elt	Line	Int	K	Kr	W%	A%
C	Ka	624.8	0.5905	0.2181	50.34	61.74
N	Ka	26.9	0.0256	0.0094	9.59	10.09
O	Ka	111.8	0.1074	0.0396	27.19	25.04
Fe	Ka	83.7	0.1111	0.0410	4.83	1.27
Cu	Ka	115.7	0.1655	0.0611	8.04	1.86
			1.0000	0.3693	100.00	100.00

**Figure 8:**
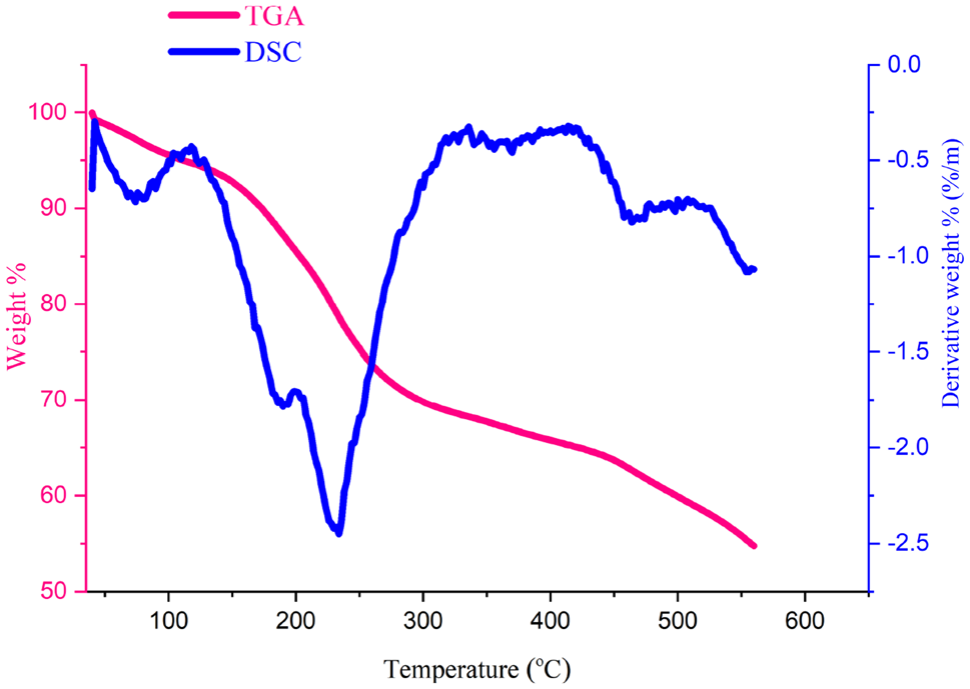
TGA and DSC curves of GO@Fe_3_O_4_@TRMS@HBPB@Cu between 40 °C to 560 °C.

The thermogravimetric curves of GO@Fe_3_O_4_@TRMS@HBPB@Cu are shown in Fig. 8. The analysis was performed at a rate of 10 °C per min in the temperature range of 40–550. Examining the graph of thermal resistance analysis shows three stages of reduction. that the first reduction in the range of initial heating is observed at a temperature of 130 °C, which is due to the loss of water content and solvents in the sample. The second reduction was done at about 230–300 °C, which corresponding to the decomposition of the ligand in the compound. A slow and constant mass reduction occurs in the temperature range of 300–430 °C, which can be related to the removal of copper metal, and the next mass reduction is after the temperature of 450 °C, which is related to the decomposition of the catalyst. The DSC curve also confirms the TGA curve.

**Figure 9:**
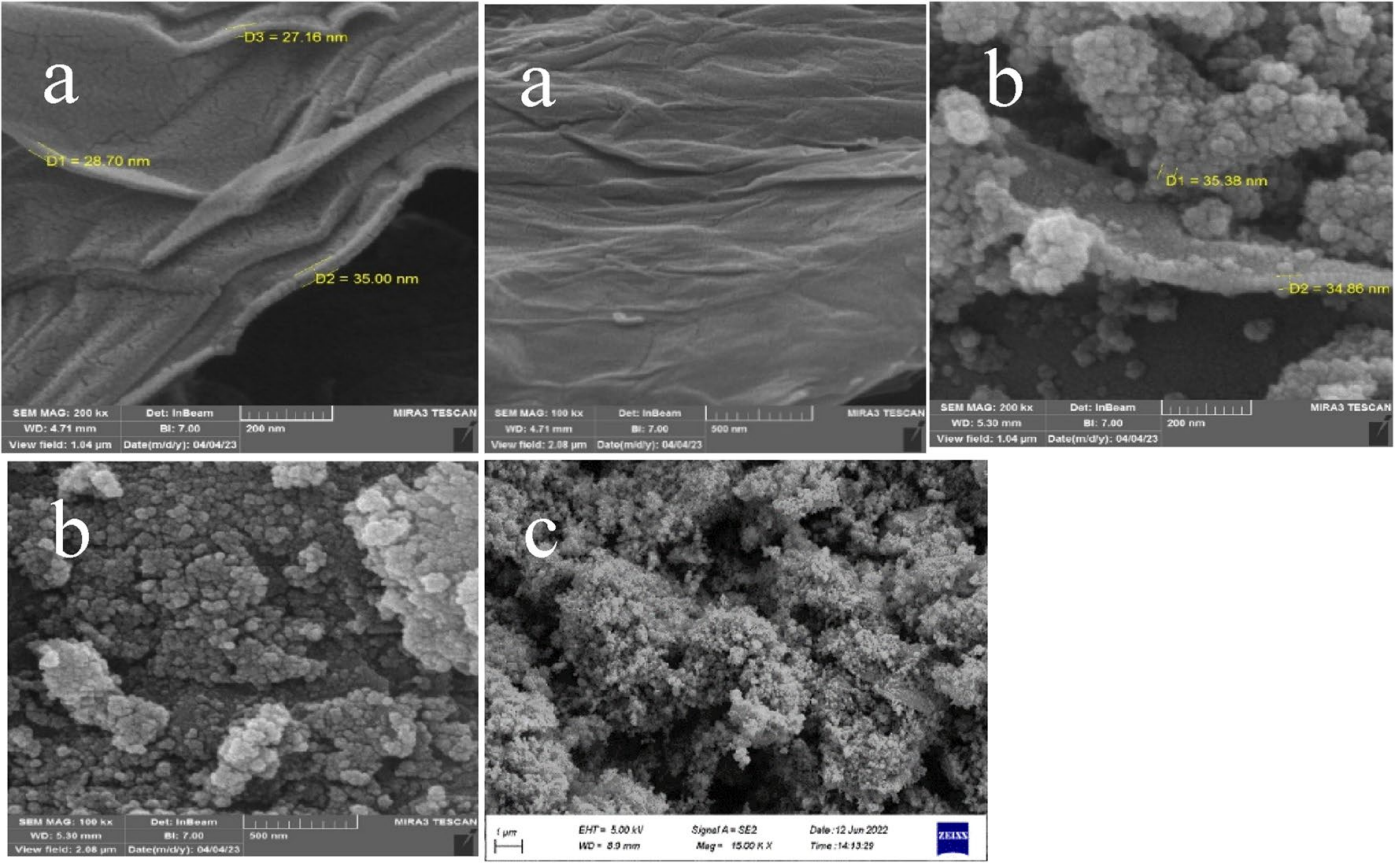
FESEM images of (**a**) GO, (**b**) GO@Fe_3_O_4_@TRMS, and (**c**) GO@Fe_3_O_4_@TRMS@HBPB@Cu.

The quality of the synthesized composite depends on the filler nanoparticles and their dispersion in the composite matrix, which leads to the improvement of its chemical, physical, thermal and electrochemical properties. FESEM was used to investigate the morphology, structure and size of the catalyst particles, SEM images with lower magnification showed the edges and wrinkles of GO nanosheets (Fig. 9, part a). In the (b) part, the white spherical dots are related to iron nanoparticles and the gray particles are related to the placement of 3-chloropropyltrimethoxysilane on magnetic graphene. As it is clear from part (c), copper nanoparticles with high and uniform dispersion are placed on the surface of the catalyst.

**Figure 10:**
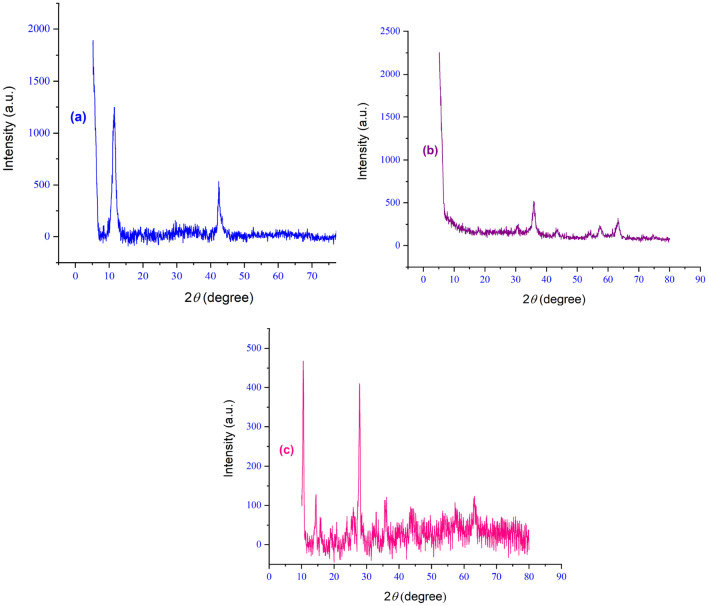
XRD pattern of (**a**) GO, (**b**) GO@Fe_3_O_4_, and (**c**) GO@Fe_3_O_4_@TRMS@HBPB@Cu nanocomposites.

 Using XRD analysis, the structural and crystalline properties of the manufactured catalyst were investigated, as it is clear from spectrum (a) of Fig. 10, the peaks of 30.93º, 10.58º and 22.39º are related to graphene, The sharp peak corresponding to magnetite appears at 34.97◦, 41.53◦, 50.41◦, 62.81◦, 67.29◦ and 74.33◦. Part (c) shows the XRD of the final stage of the catalyst, in addition to the peaks related to the previous stages, the peaks related to copper metal have also been observed, that peaks 43.35º, 55º and 72º are characteristic of copper metal, which confirms the stabilization of copper metal on the surface of the composite.

**Figure 11:**
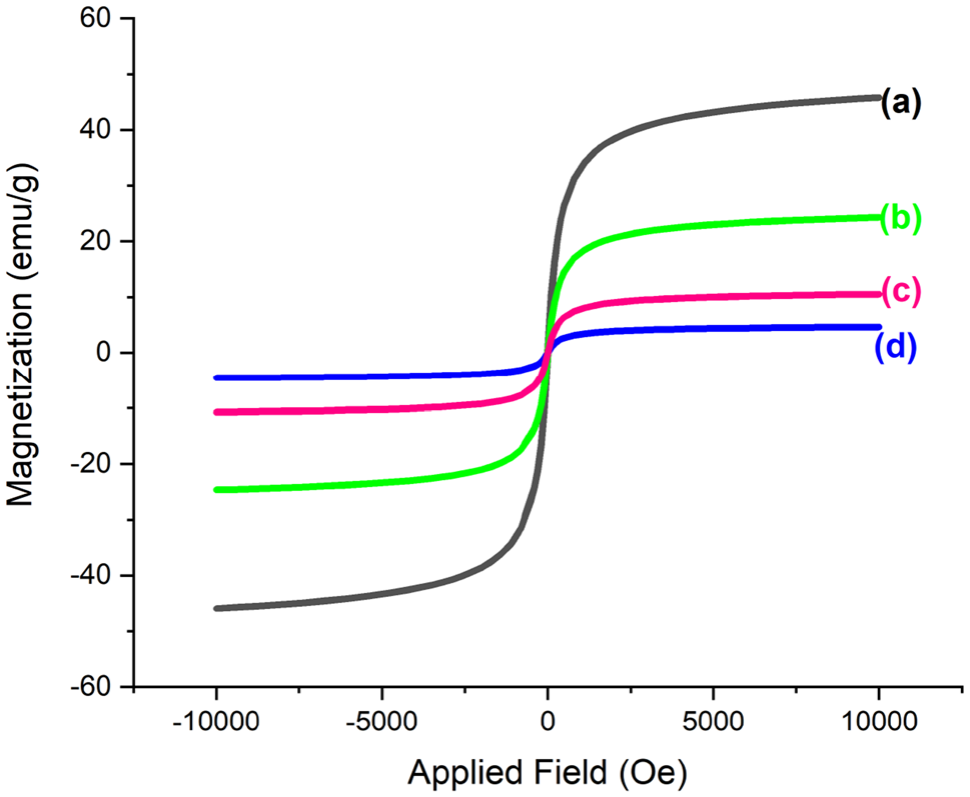
VSM curves of (**a**) GO@Fe_3_O_4_, (**b**) GO@Fe_3_O_4_@TRMS, (**c**) GO@Fe_3_O_4_@TRMS@HBPB and (**d**) GO@Fe_3_O_4_@TRMS@HBPB@Cu.

**Figure 12:**
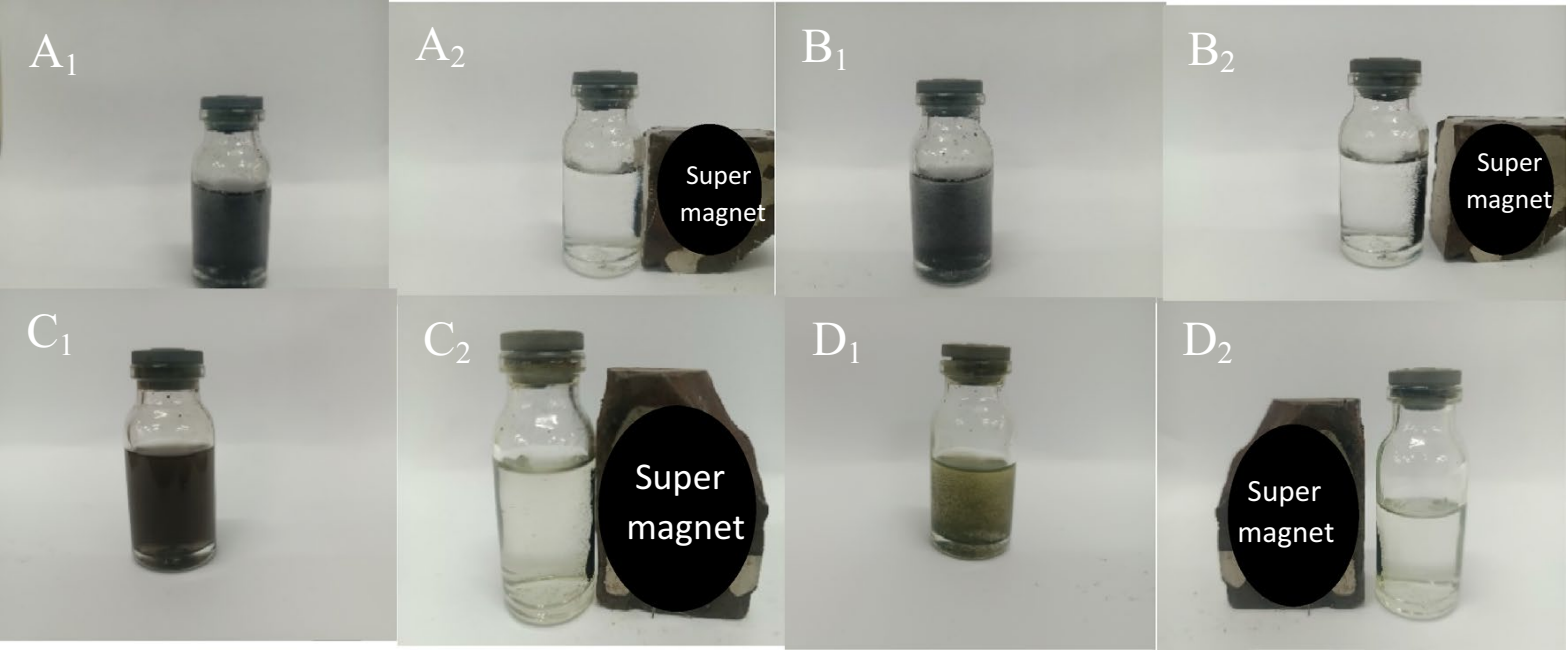
Separation images of of GO@Fe_3_O_4_ (A_1_and A_2_), GO@Fe_3_O_4_@TRMS (B_1_and B_2_), GO@Fe_3_O_4_@TRMS@HBPB (C_1_and C_2_) and GO@Fe_3_O_4_@TRMS@HBPB@Cu (D_1_and D_2_) intermediates from aqueous solution under external magnetic field.

The magnetic property of the synthesized catalyst is important due to its easy separation from the reaction mixture and easy recycling. Therefore, the magnetic value of the samples was determined by using Vibrating Sample Magnetometer (VSM) analysis. Figure 11 shows the VSM curves of (a) GO@Fe_3_O_4_, (b) GO@Fe_3_O_4_@TRMS, (c) GO@Fe_3_O_4_@TRMS@HBPB and (d) synthesized GO@Fe_3_O_4_@TRMS@HBPB@Cu nanocomposite. As can be seen from the figure, the magnetic property has decreased with the placement of the layers in order, and this indicates the optimal placement of the layers. The magnetic value of the GO@Fe_3_O_4_ sample is about 40, and when 3-chloropropyltrimethoxysilane is placed on GO@Fe_3_O_4_, the magnetic value reaches about 20, and when the ligand is placed on the previous step, the magnetic value decreases to 10, and in the last step, with the stabilization of copper metal on GO@Fe_3_O_4_@TRMS@HBPB, the magnetic value reaches 4, and these nanoparticles are easily separated from the reaction medium by a super magnet. Also, Fig. 12 shows the images related to the separation of the nano-catalyst intermediates by the super magnet, which confirms the VSM analysis and shows the easy recovery of the nano-catalyst.

### Application of GO@Fe_3_O_4_@TRMS@HBPB@Cu(II) NPs

To reveal the performance of this GO@Fe_3_O_4_@TRMS@HBPB@Cu catalyst, a three-component one-pot synthesis of quinazoline derivatives was performed from 5-chloro-2-aminophenone, ammonium acetate and a wide range of benzaldehydes with acceptor and donor groups. To investigate the main effective factors in the synthesis of quinazoline derivatives, the optimization of the effective parameters was evaluated (Table 2). For this purpose, 5-chloro-2-aminobenzophenone, ammonium acetate and 5-chlorobenzaldehyde were used in different conditions of different solvents, different temperatures and in the presence and absence of the catalyst. The best performance was obtained in the presence of 50 mg of catalyst, without solvent and at a temperature of 45 °C. The reaction of the model was investigated in acetonitrile, ethanol, methanol and water solvents, and it was found that the different solvents used have different effects on the synthesis of the product. As it is known from Table 2, ethanol and methanol solvents have a better effect than other solvents. Also, the examination of the reaction in the presence and absence of the catalyst showed that in the absence of the catalyst, the yield of the product after a few hours was small, while in the presence of the catalyst, the yield of the product increased significantly, which indicates the high performance of the catalyst. At the same time, different amounts of the catalyst were also investigated, as can be deduced from the table, the efficiency increases and the reaction time decreases with the increase in the amount of the catalyst from 10 to 50 mg, higher efficiency was not observed by increasing the amount of catalyst from 50, which may be due to the rebound effect of the catalyst. And it is also possible to increase the catalyst from a certain amount to disturb the collision between raw materials, which will reduce the yield of the product. Therefore, the optimal amount of catalyst was obtained 50 mg. In addition, different temperatures were investigated, it is obvious that the efficiency increases with the increase in temperature, but with the increase in temperature from 45 °C to 55 °C, no change in efficiency was observed, and with the increase in temperature from 55 °C to 70 °C, we encountered a decrease in product efficiency, which is the probability of reaction reversal, the best conditions were obtained at 45 °C. Table 3 shows the investigation of the model reaction in the presence of catalyst intermediates. As it is clear from the table, graphene oxide gives a trace product in half an hour, with the magnetization of graphene oxide, the yield of the product increases dramatically. No significant change was observed when 3-chlorotrimethoxysilane was placed on the surface of magnetized graphene oxide, but when the ligand was placed on its surface, the yield of the product increased, and the highest yield was obtained in the presence of copper fixed to the ligand placed on the magnetized graphene oxide surface. It can be concluded that the presence of iron metal in addition to copper metal causes the progress of the reaction. Also, a hot filtration test was done to check the efficiency of the catalyst. The model reaction was carried out in the presence of 50 mg of catalyst under optimal conditions. At the time of 11-min, which is half of the full reaction time, the catalyst was recovered by adding chloroform to the reaction container and the product yield was 74%. In another test tube, the model reaction was done that the catalyst was recycled in the middle of the reaction, continued without the presence of the catalyst, which product yield was 76% in 22-min, which did not show a significant increase, that confirmed the effectiveness of the catalyst in promoting the reaction. The optimal condition was used for the synthesis of different derivatives of quinazolines (Table 4).

**Table 2:** Optimization of the catalytic activity of GO@Fe_3_O_4_@TRMS@HBPB@Cu NPs in quinazoline synthesis.

Entry	Catalyst (mg)	Solvent	Temperature (°C)	Time (min)	Yield (%)
1	–	–	45	60	Trace
2	10	–	45	25	61
3	20	–	45	25	68
4	35	–	45	25	86
5	50	–	45	22	95
6	60	–	45	22	88
7	50	–	55	25	95
8	50	–	70	25	86
9	50	EtOH	reflux	30	83
10	50	MeOH	reflux	30	72
11	50	EtOH/H_2_O	reflux	30	68
12	50	CH_3_CN	reflux	30	35
13	50	H_2_O	reflux	30	Trace

^a^Reaction conditions: aryl benzaldehyde (1 mmol), 5-chloro-2-aminobenzophenone (1 mmol), and ammonium acetate (2.5 mmol, 0.231 g).

^b^Isolated yield.

**Table 3:** Comparison of the catalytic activity of GO@Fe_3_O_4_@TRMS@HBPB@Cu catalyst and its related intermediates.

Entry	Catalyst	Time	Yield (%)^b^
1	GO	30	Trace
2	GO@Fe_3_O_4_	25	60
3	GO@Fe_3_O_4_@TRMS	25	52
4	GO@Fe_3_O_4_@TRMS@HBPB	25	68
5	GO@Fe_3_O_4_@TRMS@HBPB@Cu	22	95

^a^Reaction conditions: aryl benzaldehyde (1 mmol), 5-chloro-2-aminobenzophenone (1 mmol), and ammonium acetate (2.5 mmol, 0.231 g).

^b^Isolated yield.

**Table 4:**
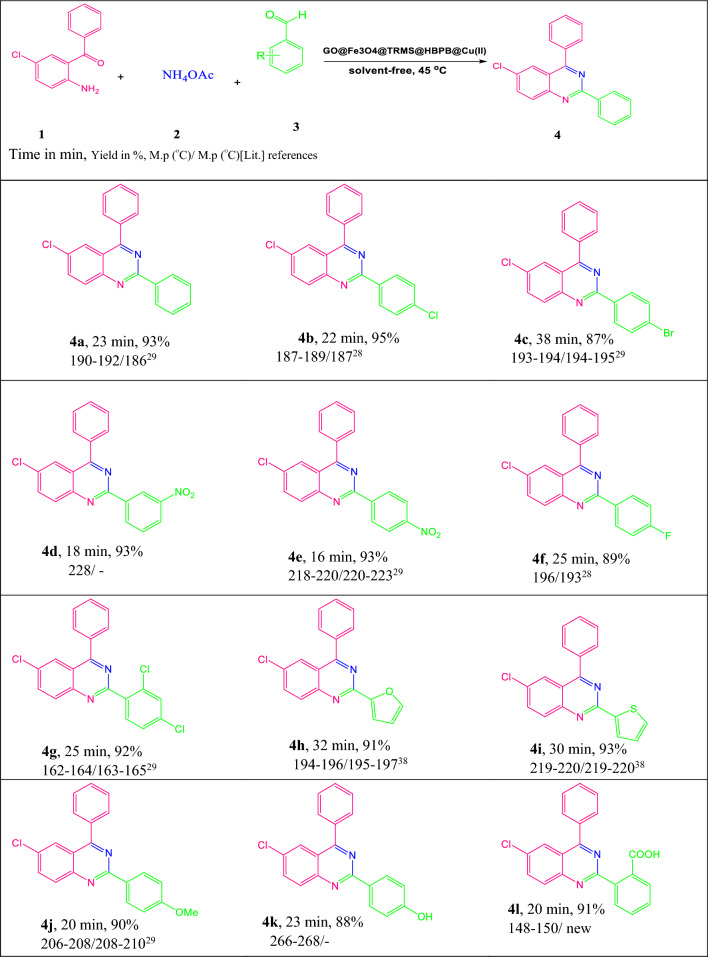
Synthesis of quinazoline derivatives under optimum conditions

^a^Reaction conditions: aryl benzaldehyde (1 mmol), 5-chloro-2-aminobenzophenone (1 mmol), and ammonium acetate (2.5 mmol, 0.231 g).

^b^Isolated yield.

Further, to prove the efficiency of the novel catalyst and the scope of the method, with optimal conditions, various quinazoline derivatives with a wide range of aldehydes, ammonium acetate, and 5-chloro-2-aminobenzophenone were investigated in the presence of 50 mg of catalyst in solvent-free conditions and at a temperature of 45 °C, and the results are collected in Table 4. The results showed high performance of the catalyst and short reaction time. It can be seen from Table 4 that the giving and killing effects of groups on benzaldehyde do not significantly affect the yield of the desired product. But with electron acceptor groups on benzaldehyde, products are made with less reaction time, but donating groups need more time.

**Figure 13:**
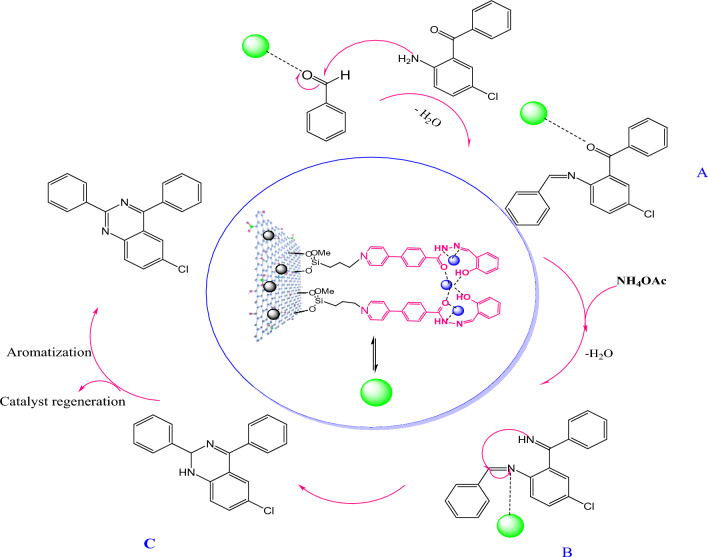
Suggested mechanism for the catalytic role of GO@Fe_3_O_4_@TRMS@HBPB@Cu NPs in the quinazoline synthesis reaction.

The proposed mechanism for the catalytic role of GO@Fe_3_O_4_@TRMS@HBPB@Cu(II) nanoparticles in the synthesis reaction of quinazolines is presented in Fig. 13. As it is clear from the scheme, the oxygen and nitrogen atoms in the structures of 5-chloro-2-aminobenzophenone, play a key role in this process by creating chelation with chelated copper ions. They reduce the reaction time by activating the carbonyl groups of benzaldehyde and 5-chloro-2-aminobenzophenone. As can be seen, first, from the reaction between activated aryl aldehydes by nano-catalyst and 5-chlorobenzophenone, intermediate A is formed. Intermediate B is formed by the reaction between ammonium acetate and active carbonyl of intermediate A. Intermediate C was obtained by the intramolecular reaction of nitrogen attack on the double bond and the formation of a hexagonal ring. Finally, the final product was synthesized by tautomerization and aromatization, and the catalyst is magnetically recovered and reused.

An important parameter in catalytic performance is catalyst recovery. For this purpose, after the reaction of the model by adding chloroform to the reaction medium, the catalyst was removed from the reaction vessel with an external magnetic system, and after washing it several times with ethanol, it was placed in the oven at a temperature of 60 °C to dry for 24 h, and after that, it was used again in the model test, and the results showed that no significant change in its activity was observed at least four consecutive cycles, which clearly shows the stability and durability of the catalyst (Fig. 1^4^). For each run of the model reaction, the catalyst was recovered in the amount of 0.044 g, the second time 0.041 g, the third time 0.037 g and the fourth time 0.034 g. Figure 15is the FTIR image of the recovery catalyst after four stages of the model reaction, which shows there is no significant change with the FTIR spectrum of the initial catalyst. Also, Figs. 16 and 17 show the EDX and XRD analyzes of the recycled catalyst, respectively. The EDX analysis shows the presence of all the elements in the catalyst such as carbon, nitrogen, oxygen, iron and copper, which indicates the stability of the catalyst (Fig. 16). The XRD pattern is similar to the XRD of the original catalyst, which shows the structure of the catalyst has remained stable. These analyzes show an acceptable recycling for the GO@Fe_3_O_4_@TRMS@HBPB@Cu catalyst. Figure 18 shows the SEM images of the recycled catalyst, which was used to check the morphology and surface of the catalyst, as it is clear from the images the copper nanoparticles are uniformly and harmoniously placed on its surface, the surface of the catalyst has not changed significantly after four cycles of recycling, and the size of the particles is in the nano range, and it shows the catalyst had an acceptable recovery.

**Figure 14:**
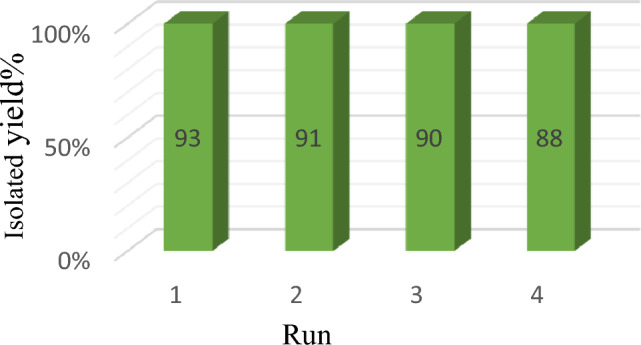
Recycling of the GO@Fe_3_O_4_@TRMS@HBPB@Cu NPs for the reaction of the model.

**Figure 15:**
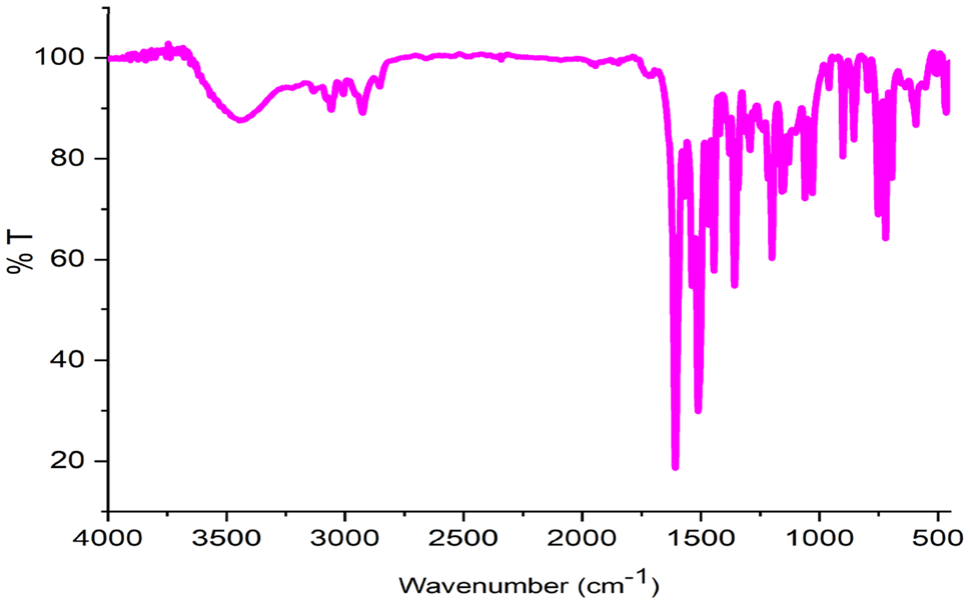
FTIR **s**pectrum of the recycled GO@Fe_3_O_4_@TRMS@HBPB@Cu NPs.

**Figure 16:**
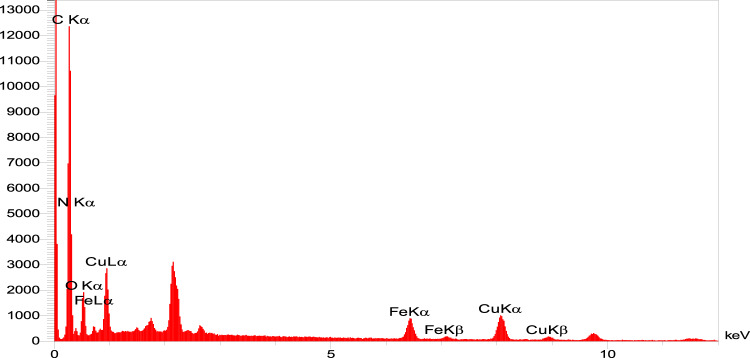
EDX spectra of recycled GO@Fe_3_O_4_@TRMS@HBPB@Cu.

**Figure 17:**
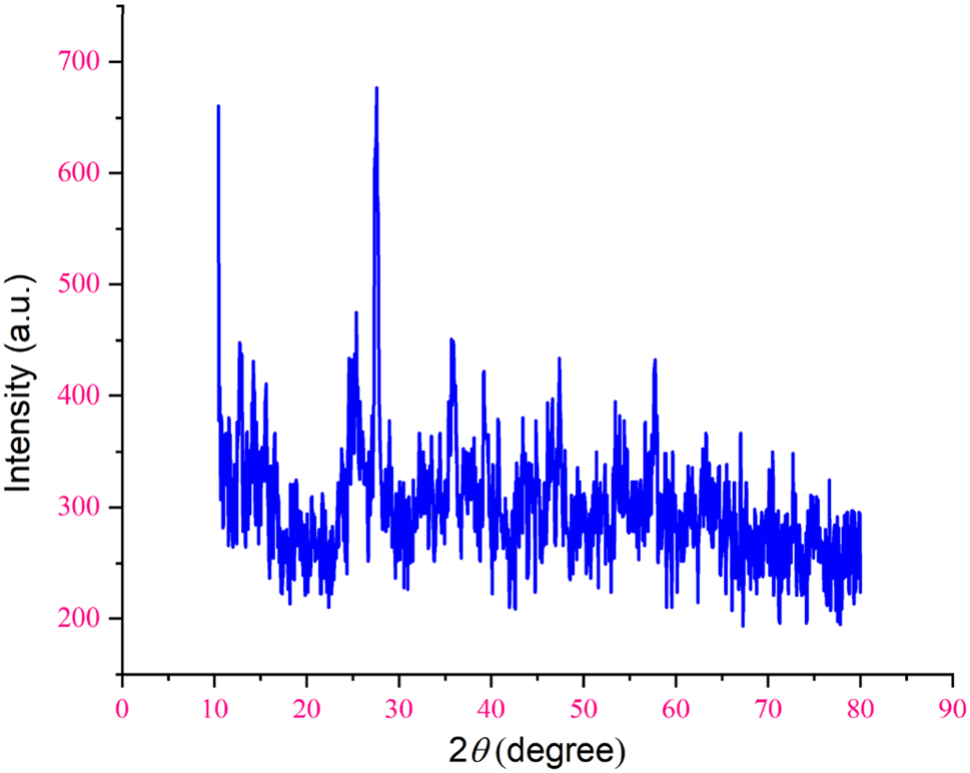
XRD pattern of the recycled GO@Fe_3_O_4_@TRMS@HBPB@Cu NPs.

**Figure 18:**
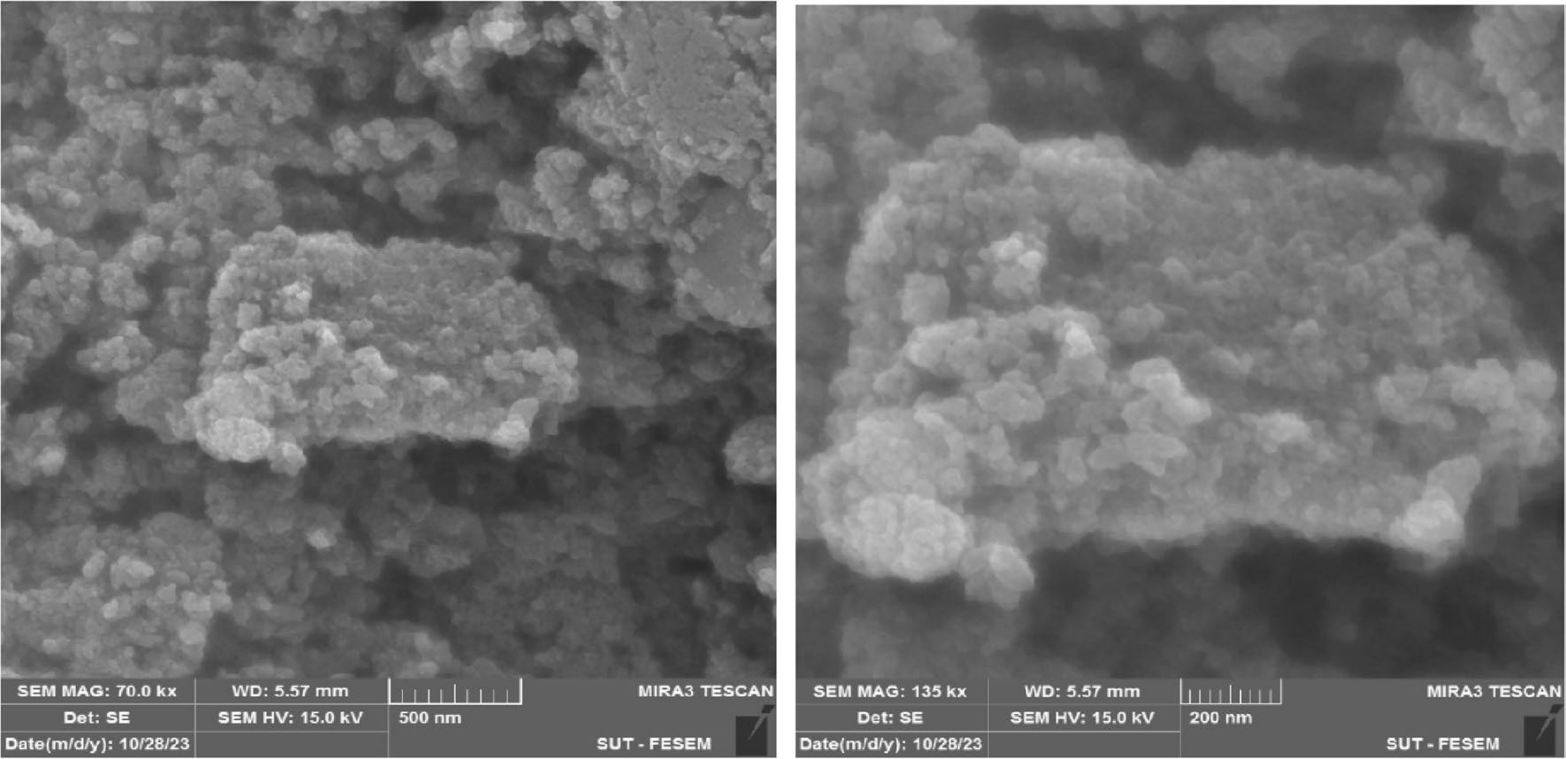
SEM patterns of the recycled GO@Fe_3_O_4_@TRMS@HBPB@Cu NPs.

The efficiency of the novel recyclable heterogeneous catalyst was compared with other reported catalysts for the synthesis of quinazolines, and the results of this comparison are summarized in Table 5. As can be seen from Table 5, the GO@Fe_3_O_4_@TRMS@HBPB@Cu catalyst as an efficient and green catalyst with reusable capability has performed the synthesis of quinazoline derivatives with high efficiency, short time and low temperature.

**Table 5:** Comparison of the performance of the GO@Fe_3_O_4_@TRMS@HBPB@Cu catalyst with several other catalysts in synthesis quinazoline derivatives.

Entry	Reaction conditions	Time (min)	Yield (%)	Lit
1	Fe_3_O_4_@SiO_2_-PrNH_2_-Fe^3+^, Solvent-free, 60 °C	240	86	^48^
2	TBBDA or PBBS, Ethanol, 45 °C	180	88	^38^
3	maltose–DMU–NH_4_Cl, Air, 90 °C	150	93	^29^
4	triethanolammonium-2,2,2-trichloroacetate (TEATCA), 80°C	15	85	^49^
5	Bmim[FeCl_4_], Solvent-free, 40 °C	150	94	^40^
6	[Hmim]TFA, Solvent-free, 80°C	120	94	^28^
7	GO@Fe_3_O_4_@TRMS@HBPB@Cu(II), 45 °C	22	95	This work

## Conclusion

Quinazoline derivatives are an important group of heterocyclic compounds that are of interest to researchers due to their medicinal properties, and their more effective synthetic methods are of great importance. For this reason, we have attempted to design a high-performance, recyclable, heterogeneous catalyst and use it for the one-pot three-component synthesis of quinazolines. The novel catalyst was synthesized from copper acetate immobilized on the base of magnetized and modified graphene oxide, and its structure was checked and confirmed by various analyses such as FTIR, XRD, EDX, MAPPING, TGA/DSC, VSM and FESEM. On the one hand, the high performance of the catalyst through the interaction of chelated copper stabilized on the catalyst with the carbonyl and nitrogen groups of the raw materials leads to the easy synthesis of the product, and on the other hand, due to the magnetic properties of GO@Fe_3_O_4_@TRMS@HBPB@Cu nanoparticles, they were easily separated from the reaction mixture and recycled. It seems that iron metal, not only recovering the catalyst, but also helps the progress of the reaction. The control experiments were well monitored by using 50 mg of nano-catalyst for temperature of 45 °C, the products were obtained with high efficiency (Table 4). In the end, the recyclability of the catalyst was examined, it was recycled 4 times and no significant change in its efficiency was observed (Fig. 1^4^).

### Supplementary Information

Supplementary Information.

## Data Availability

All data generated or analyzed during this study are included in the supplementary information file.
