# Supplementary material for: Green synthesis of quinazoline derivatives using a novel recyclable nano-catalyst of magnetic modified graphene oxide supported with copper
Source: Sci Rep. 2023 Nov 28;13:20958. doi: 10.1038/s41598-023-48120-6 (PMC10684527; doi:10.1038/s41598-023-48120-6)
Supplement: Supplementary file 1 — Supplementary Information. [file 41598_2023_48120_MOESM1_ESM.docx]

**Green synthesis of quinazoline derivatives using a novel recyclable nano-catalyst of magnetic modified graphene oxide supported with copper**

**(Scientific Reports)**

Sarieh Momeni, Ramin Ghorbani-Vaghei*

*Department of Organic Chemistry, Faculty of Chemistry, Bu-Ali Sina University, Hamedan, 6517838683, Iran*

**Corresponding author; E-mail:* [*rgvaghei@yahoo.com*](mailto:rgvaghei@yahoo.com) *&* [*ghorbani@basu.ac.ir*](mailto:ghorbani@basu.ac.ir)

| ***Table of Contents*** | ***Page number*** |
| --- | --- |
| ***Spectra data*** |  |
| 6-chloro-2,4-diphenylquinazoline (4a) | 4 |
| 6-chloro-2-(4-chlorophenyl)-4-phenylquinazoline (4b) | 6 |
| 2-(4-bromophenyl)-6-chloro-4-phenylquinazoline (4c) | 10 |
| 6-chloro-2-(3-nitrophenyl)-4-phenylquinazoline (4d) | 12 |
| 6-chloro-2-(4-nitrophenyl)-4-phenylquinazoline (4e) | 14 |
| 6-chloro-2-(4-fluorophenyl)-4-phenylquinazoline (4f) | 15 |
| 6-chloro-2-(2,4-dichlorophenyl)-4-phenylquinazoline (4g) | 18 |
| 6-chloro-2-(furan-2-yl)-4-phenylquinazoline (4h) | 20 |
| 6-chloro-4-phenyl-2-(thiophen-2-yl)quinazoline (4i)  6-chloro-2-(4-methoxyphenyl)-4-phenylquinazoline (4j)  4-(6-chloro-4-phenylquinazolin-2-yl)phenol (4k)  2-(6-chloro-4-phenylquinazolin-2-yl)benzoic acid (4l) | 23  25  28  31 |
| Figure 1. The FTIR spectrum of 6-chloro-2,4-diphenylquinazoline (4a) | 4 |
| Figure 2. The ^1^H NMR (500 MHz) spectrum of 6-chloro-2,4-diphenylquinazoline in CDCl_3_ solvent (4a) | 5 |
| Figure 3. The CNMR (101 MHz) spectrum of 6-chloro-2,4-diphenylquinazoline in CDCl_3_ solvent (4a) | 6 |
| Figure 4. The FTIR spectrum of 6-chloro-2-(4-chlorophenyl)-4-phenylquinazoline (4b) | 7 |
| Figure 5. The ^1^H NMR (500 MHz) spectrum of 6-chloro-2-(4-chlorophenyl)-4-phenylquinazoline in CDCl_3_ solvent (4b) | 7 |
| Figure 6. Magnification of the ^1^H NMR (500 MHz) spectrum of 6-chloro-2-(4-chlorophenyl)-4-phenylquinazoline in CDCl_3_ solvent (4b) | 8 |
| Figure 7. The CNMR (101 MHz) spectrum of 6-chloro-2-(4-chlorophenyl)-4-phenylquinazoline in CDCl_3_ solvent (4b) | 9 |
| Figure 8. The ^1^H NMR (250 MHz) spectrum of 6-chloro-2-(4-chlorophenyl)-4-phenylquinazoline in DMSO solvent (4b) | 10 |
| Figure 9. The FTIR spectrum of 2-(4-bromophenyl)-6-chloro-4-phenylquinazoline (4c) | 11 |
| Figure 10. The ^1^H NMR (500 MHz) spectrum of 2-(4-bromophenyl)-6-chloro-4-phenylquinazoline in CDCl_3_ solvent (4c) | 11 |
| Figure 11. The FTIR spectrum of 6-chloro-2-(3-nitrophenyl)-4-phenylquinazoline (4d) | 12 |
| Figure 12. The ^1^H NMR (250 MHz) spectrum of 6-chloro-2-(3-nitrophenyl)-4-phenylquinazoline in CDCl_3_ solvent (4d) | 13 |
| Figure 13. The CNMR (63 MHz) spectrum of 6-chloro-2-(3-nitrophenyl)-4-phenylquinazoline in CDCl_3_ solvent (4d) | 14 |
| Figure 14. The ^1^H NMR (500 MHz) spectrum of 6-chloro-2-(4-nitrophenyl)-4-phenylquinazoline in DMSO solvent (4e)  Figure 15. The FTIR spectrum of 6-chloro-2-(4-fluorophenyl)-4-phenylquinazoline (4f) | 15  16 |
| Figure 16. The ^1^H NMR (250 MHz) spectrum of 6-chloro-2-(4-fluorophenyl)-4-phenylquinazoline in DMSO solvent (4f) | 17 |
| Figure 17. The CNMR (63 MHz) spectrum of 6-chloro-2-(4-fluorophenyl)-4-phenylquinazoline in DMSO solvent (4f) | 18 |
| Figure 18. The ^1^H NMR (250 MHz) spectrum of 6-chloro-2-(2,4-dichlorophenyl)-4-phenylquinazoline in CDCl_3_ solvent (4g) | 19 |
| Figure 19. The CNMR (63 MHz) spectrum of 6-chloro-2-(2,4-dichlorophenyl)-4-phenylquinazoline in CDCl_3_ solvent (4g) | 20 |
| Figure 20. The FTIR spectrum of 6-chloro-2-(furan-2-yl)-4-phenylquinazoline (4h) | 21 |
| Figure 21. The ^1^H NMR (250 MHz) spectrum of 6-chloro-2-(furan-2-yl)-4-phenylquinazoline in CDCl_3_ solvent (4h) | 21 |
| Figure 22. The ^1^H NMR (250 MHz) spectrum of 6-chloro-2-(furan-2-yl)-4-phenylquinazoline in DMSO solvent (4h) | 22 |
| Figure 23. The CNMR (63 MHz) spectrum of 6-chloro-2-(furan-2-yl)-4-phenylquinazoline in CDCl_3_ solvent (4h) | 23 |
| Figure 24. The ^1^H NMR (250 MHz) spectrum of 6-chloro-4-phenyl-2-(thiophen-2-yl)quinazoline in CDCl_3_ solvent (4i) | 24 |
| Figure 25. The CNMR (63 MHz) spectrum of 6-chloro-4-phenyl-2-(thiophen-2-yl)quinazoline in CDCl_3_ solvent (4i)  Figure 26. The FTIR spectrum of 6-chloro-2-(4-methoxyphenyl)-4-phenylquinazoline (4j)  Figure 27. The 1H NMR (250 MHz) spectrum of 6-chloro-2-(4-methoxyphenyl)-4-phenylquinazoline in CDCl3 solvent (4j)  Figure 28. Magnification of the 1H NMR (250 MHz) spectrum of 6-chloro-2-(4-methoxyphenyl)-4-phenylquinazoline in CDCl3 solvent (4j)  Figure 29. The CNMR (63 MHz) spectrum of 6-chloro-2-(4-methoxyphenyl)-4-phenylquinazoline in CDCl3 solvent (4j)  Figure 30. The FTIR spectrum of 4-(6-chloro-4-phenylquinazolin-2-yl)phenol (4k)  Figure 31. The 1H NMR (250 MHz) spectrum of 4-(6-chloro-4-phenylquinazolin-2-yl)phenol in DMSO solvent (4k)  Figure 32. The 1H NMR (250 MHz) spectrum of 4-(6-chloro-4-phenylquinazolin-2-yl)phenol in CDCl3 solvent (4k)  Figure 33. The CNMR (63 MHz) spectrum of 4-(6-chloro-4-phenylquinazolin-2-yl)phenol in DMSO solvent (4k)  Figure 34. The FTIR spectrum of 2-(6-chloro-4-phenylquinazolin-2-yl)benzoic acid (4l)  Figure 35. The 1H NMR (250 MHz) spectrum of 2-(6-chloro-4-phenylquinazolin-2-yl)benzoic acid in CDCl3 solvent (4l)  Figure 36. Magnification of the 1H NMR (250 MHz) spectrum of 2-(6-chloro-4-phenylquinazolin-2-yl)benzoic acid in CDCl3 solvent (4l)  Figure 37. The CNMR (63 MHz) spectrum of 2-(6-chloro-4-phenylquinazolin-2-yl)benzoic acid in DMSO solvent (4l) | 25  26  28  27  28  29  29  30  31  32  32  33  34 |

6-chloro-2,4-diphenylquinazoline (4a)

M.p. 190-192 °C, FT-IR (KBr, ν cm-1): 3050, 1611, 1559, 1537, 1479, 1415, 1388, 1340, 1161, 704; ^1^H NMR (499 MHz, Chloroform-d) δ 8.80 – 8.63 (m, 2H), 8.13 (d, J = 2.7 Hz, 2H), 7.90 (dd, J = 6.0, 3.1 Hz, 2H), 7.85 (dd, J = 9.0, 2.5 Hz, 1H), 7.65 (dd, J = 5.9, 3.0 Hz, 3H), 7.55 (d, J = 6.3 Hz, 3H); ^13^C NMR (126 MHz, DMSO-*d*_6_) δ 167.6, 163.8, 159.6, 150.5, 137.0, 134.6, 134.0, 132.7, 130.9, 130.8, 130.3, 130.0, 128.8, 125.8, 122.1.


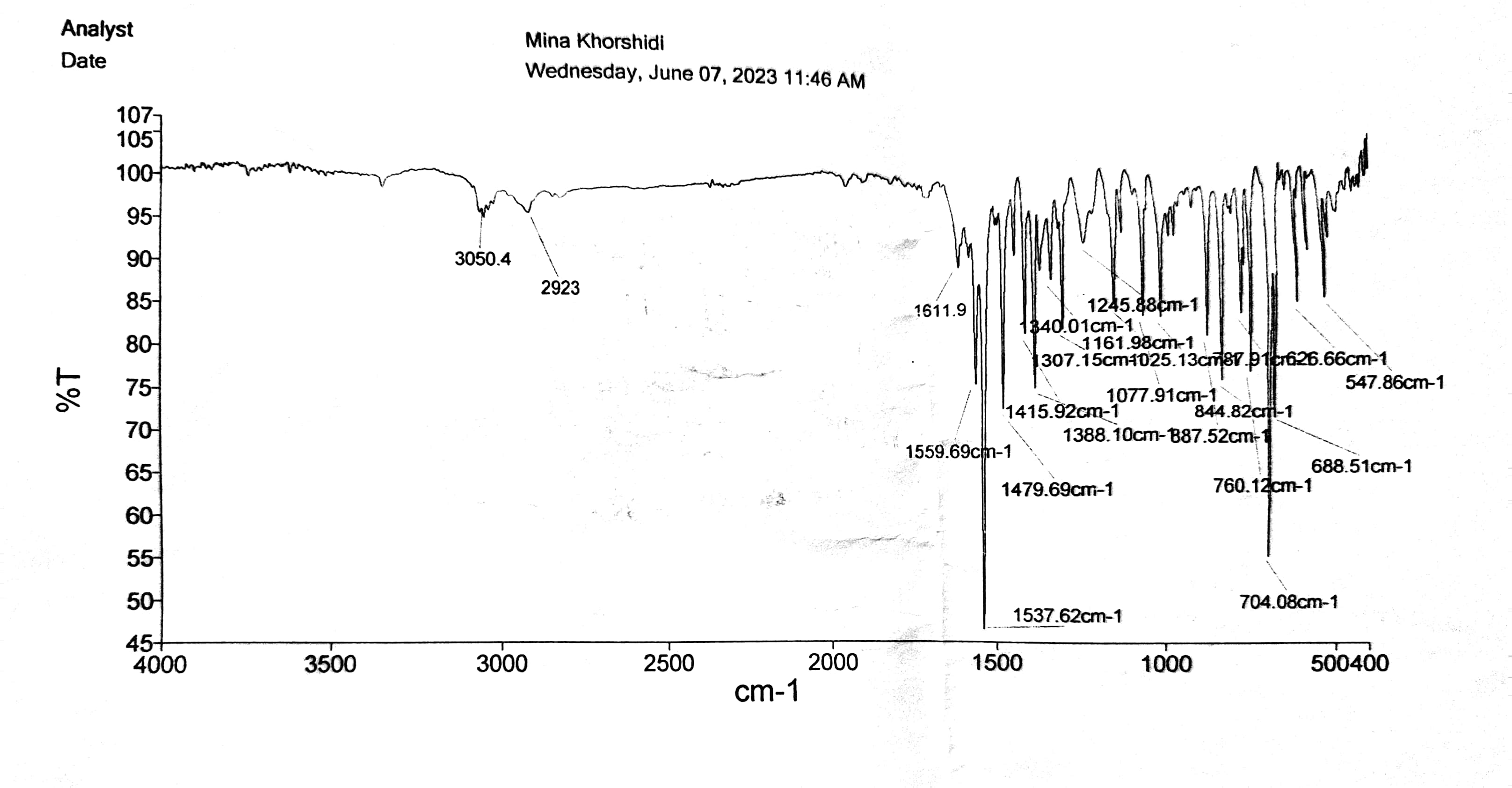


**Figure 1.** The FTIR spectrum of 6-chloro-2,4-diphenylquinazoline (4a)


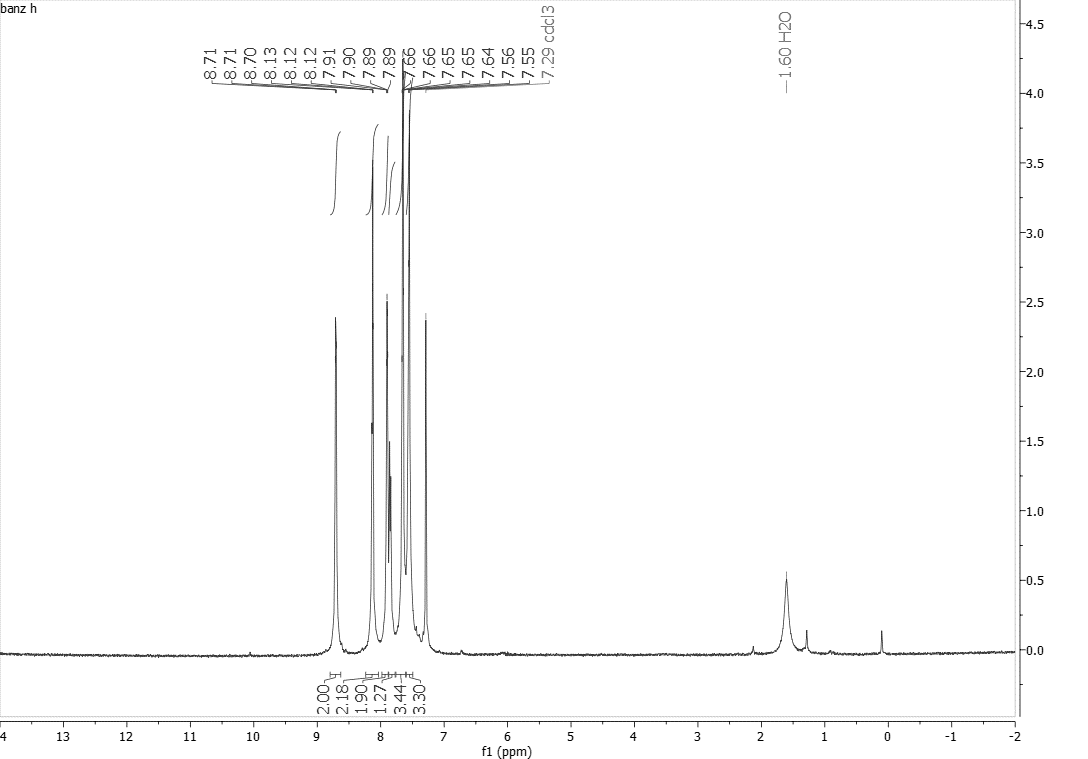


**Figure 2.** The ^1^H NMR (500 MHz) spectrum of 6-chloro-2,4-diphenylquinazoline in CDCl_3_ solvent (4a)


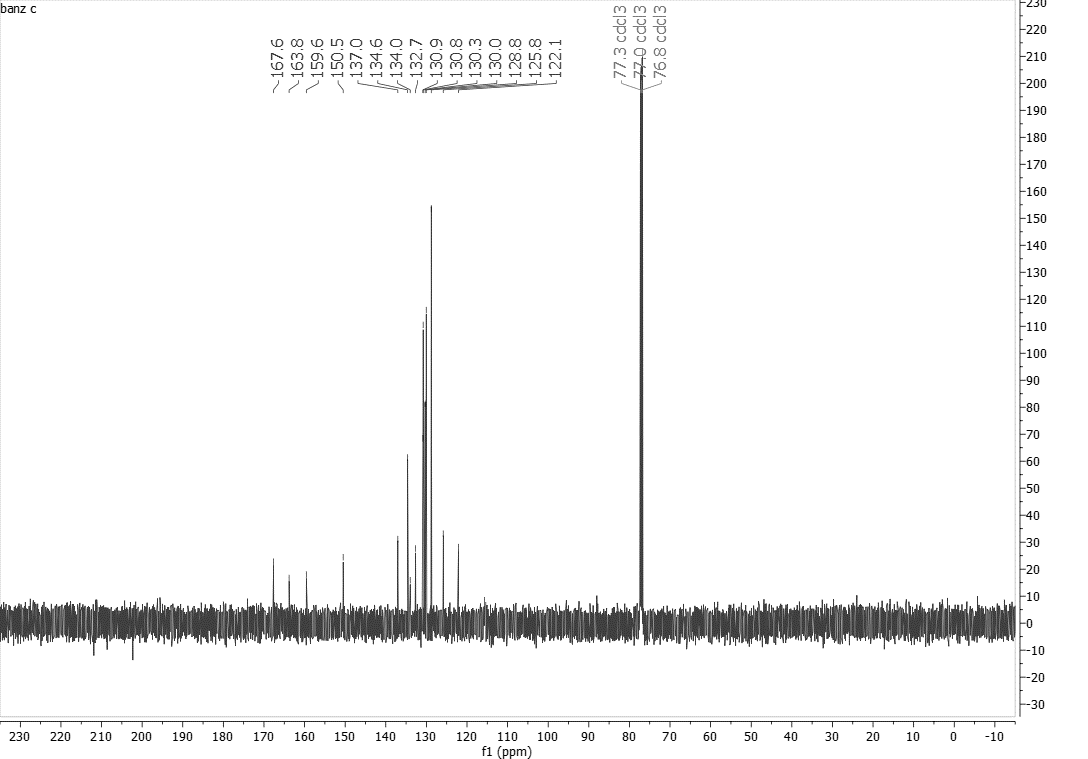


**Figure 3.** The CNMR (101 MHz) spectrum of 6-chloro-2,4-diphenylquinazoline in CDCl_3_ solvent (4a)

6-chloro-2-(4-chlorophenyl)-4-phenylquinazoline (4b)

M.p. 187-189 °C, FT-IR (KBr, ν cm-1): 3065, 1606, 1560, 1534, 1489, 1415, 1387, 1306, 1085, 1011, 837, 703; ^1^H NMR (250 MHz, Chloroform-d) δ 8.61 (d, J = 7.7 Hz, 2H), 8.06 (d, J = 9.3 Hz, 2H), 7.94 – 7.76 (m, 3H), 7.71 – 7.55 (m, 3H), 7.48 (d, J = 7.4 Hz, 2H); ^13^C NMR (63 MHz, DMSO-*d*_6_) δ 167.6, 161.6, 159.4, 150.4, 136.9, 136.2, 134.6, 132.8, 130.8, 130.2, 129.9, 128.7, 125.8, 122.1.


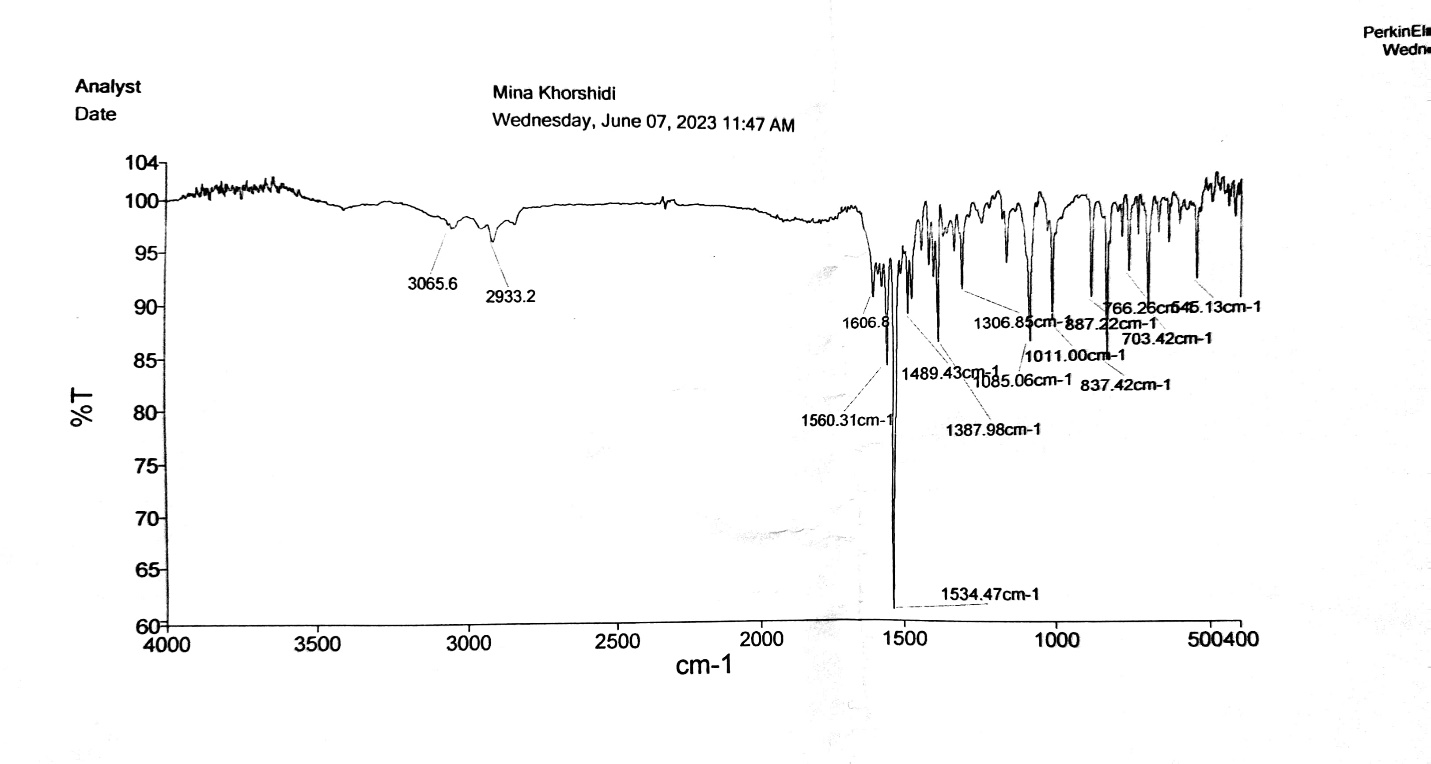


**Figure 4.** The FTIR spectrum of *6-chloro-2-(4-chlorophenyl)-4-phenylquinazoline* (4b)


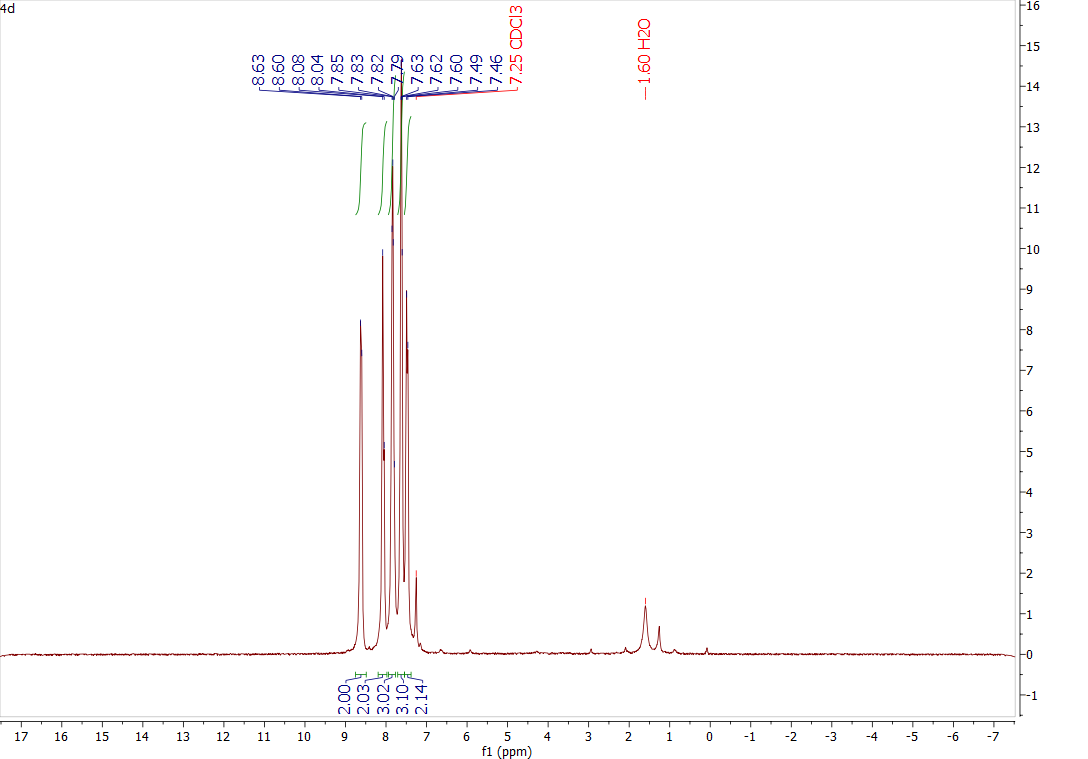


**Figure 5.** The ^1^H NMR (250 MHz) spectrum of *6-chloro-2-(4-chlorophenyl)-4-phenylquinazoline* in CDCl_3_ solvent (4b)


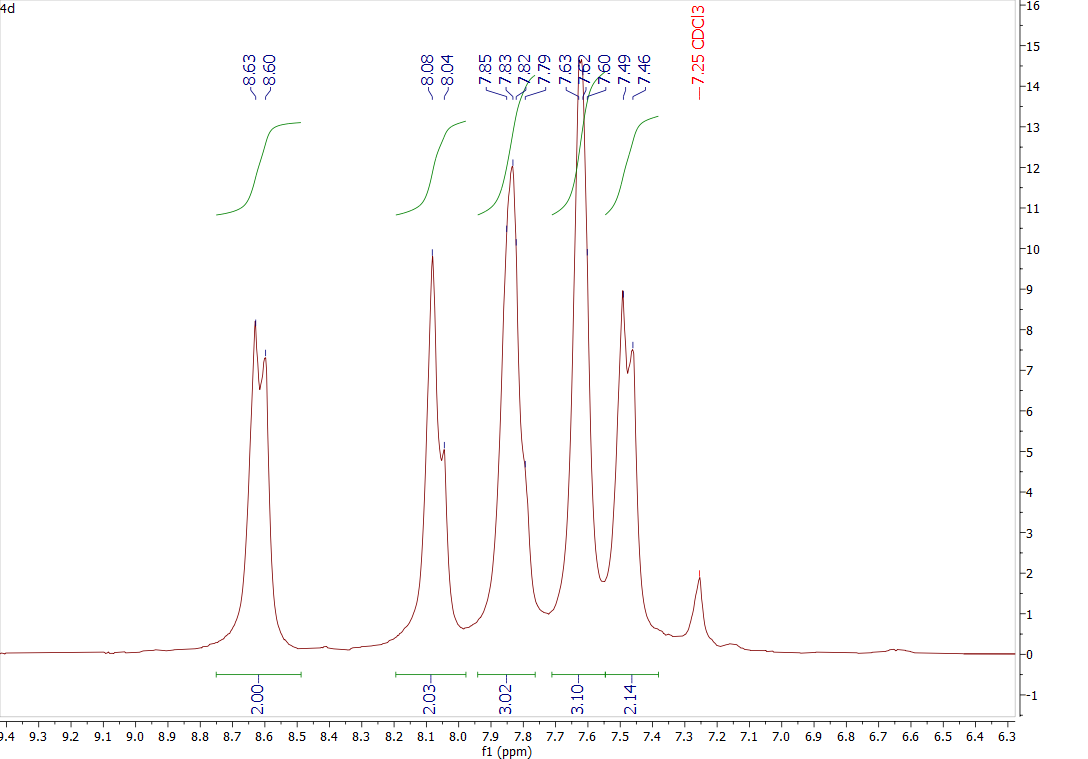


**Figure 6.** Magnification of the ^1^H NMR (250 MHz) spectrum of *6-chloro-2-(4-chlorophenyl)-4-phenylquinazoline* in CDCl_3_ solvent (4b)


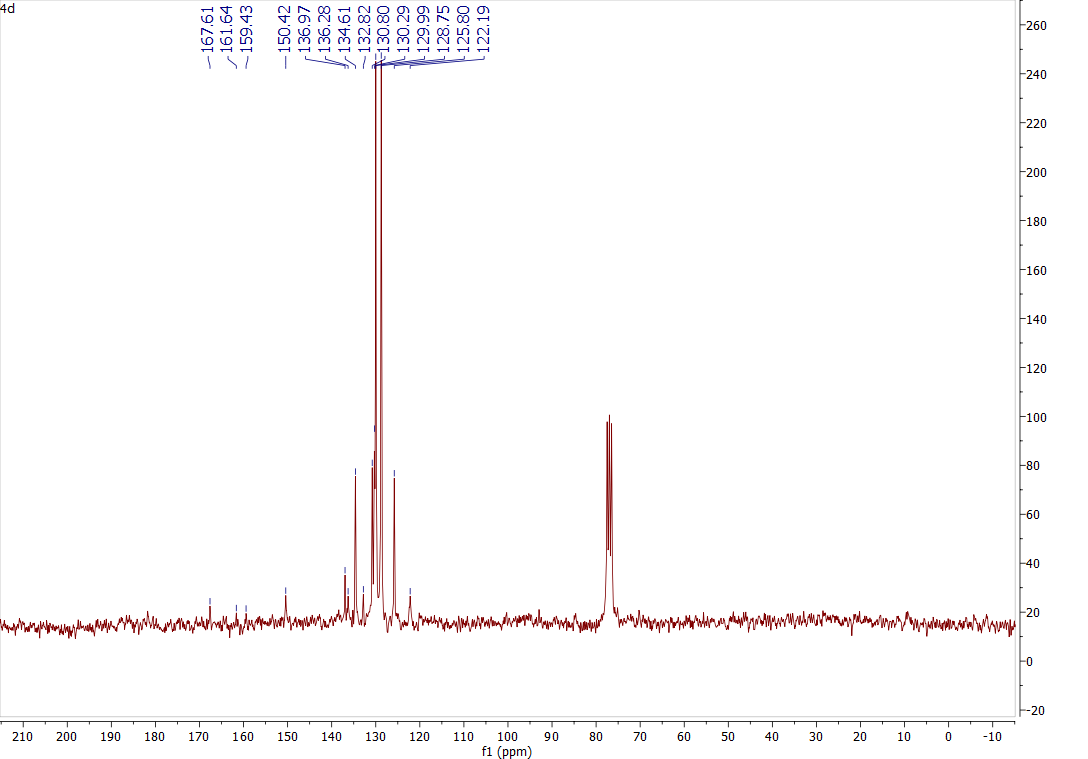


**Figure 7.** The CNMR (63 MHz) spectrum of 6-chloro-2-(4-chlorophenyl)-4-phenylquinazoline in CDCl_3_ solvent (4b)


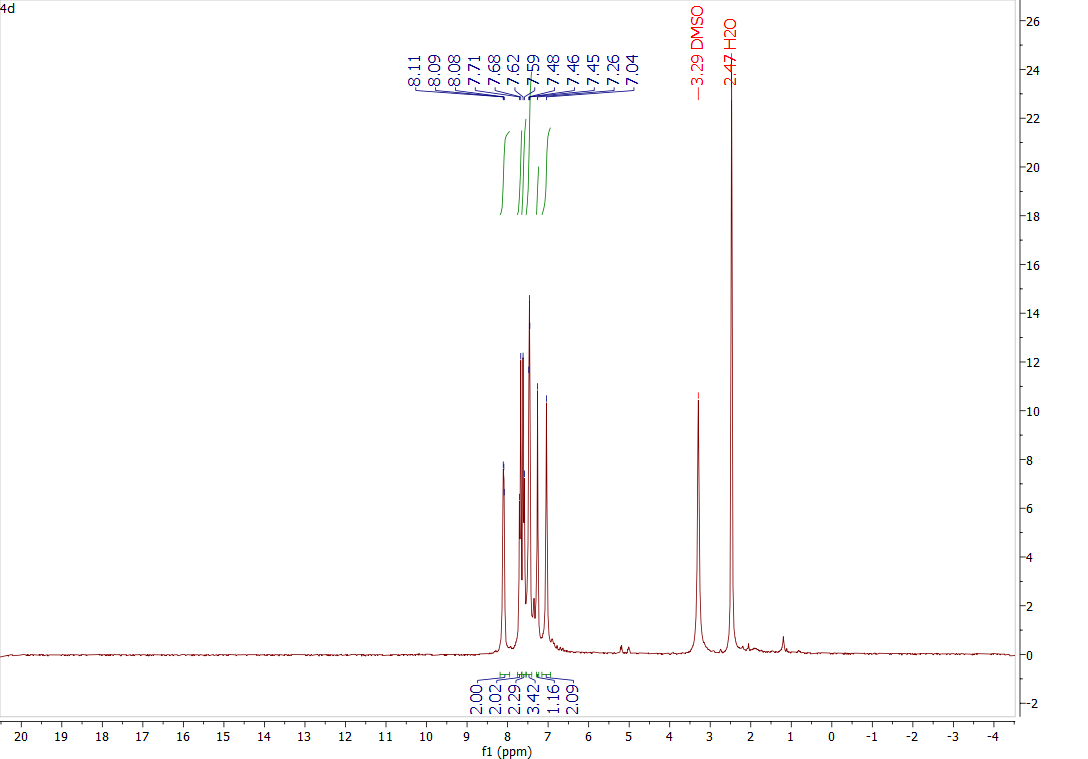


**Figure 8.** The ^1^H NMR (250 MHz) spectrum of *6-chloro-2-(4-chlorophenyl)-4-phenylquinazoline* in DMSO solvent (4b)

2-(4-bromophenyl)-6-chloro-4-phenylquinazoline (4c)

M.p. 196 °C, FT-IR (KBr, ν cm-1): 3055, 1601, 1558, 1539, 1479, 1410, 1386, 1304, 1147, 836, 775; ^1^H NMR (499 MHz, Chloroform-d) δ 8.74 – 8.68 (m, 2H), 8.10 (d, J = 10.9 Hz, 2H), 7.90 – 7.86 (m, 2H), 7.84 (d, J = 8.9 Hz, 1H), 7.67 – 7.62 (m, 3H), 7.22 (t, J = 8.5 Hz, 2H).


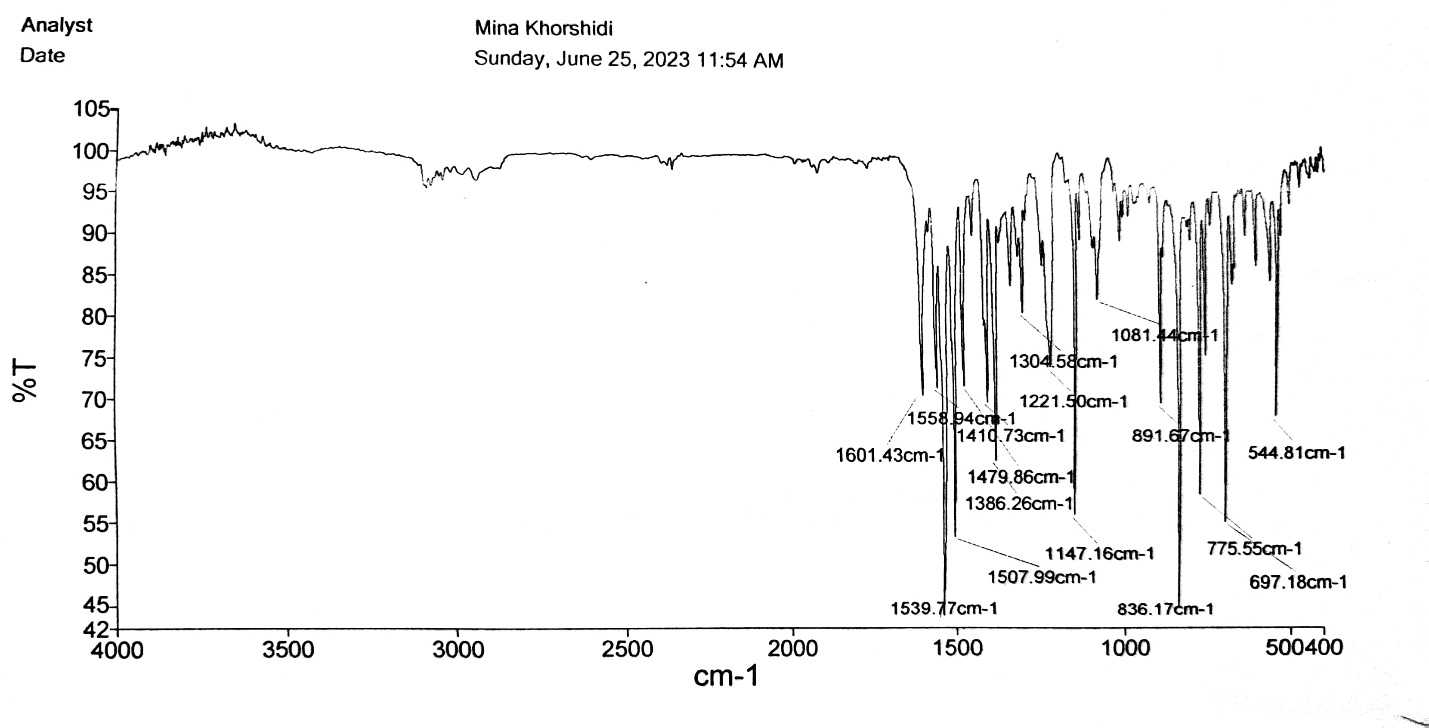


**Figure 9.** The FTIR spectrum of *2-(4-bromophenyl)-6-chloro-4-phenylquinazoline* (4c)


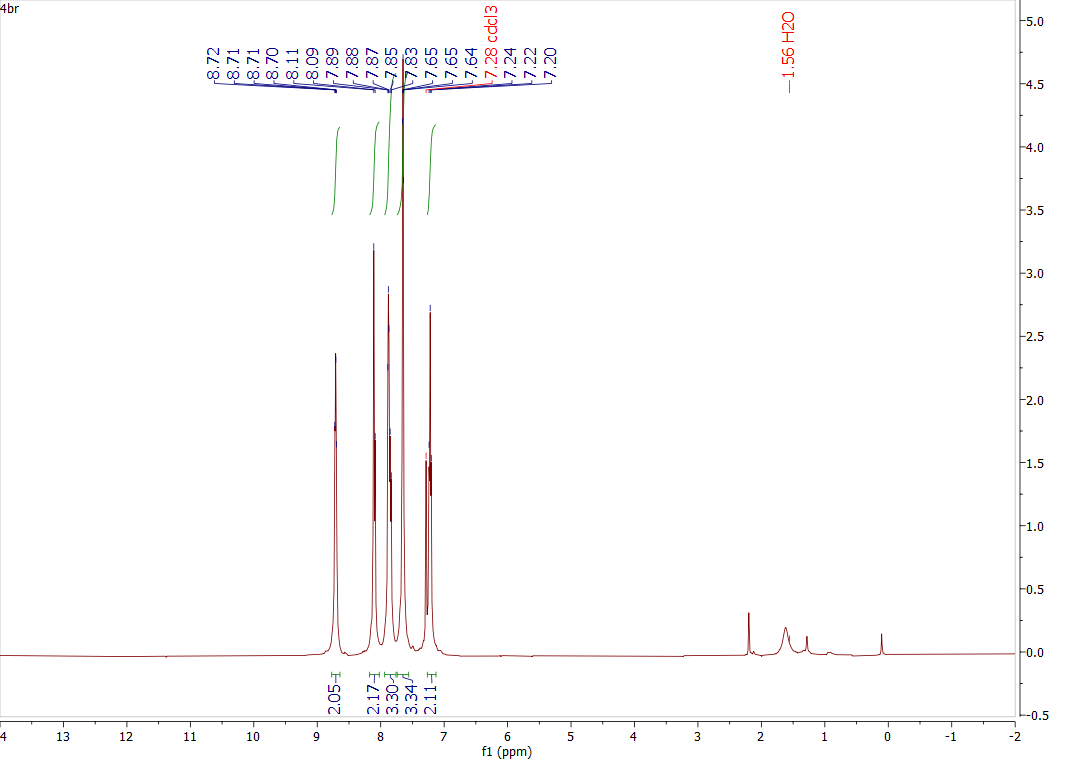


**Figure 10.** The ^1^H NMR (500 MHz) spectrum of *2-(4-bromophenyl)-6-chloro-4-phenylquinazoline* in CDCl_3_ solvent (4c)

6-chloro-2-(3-nitrophenyl)-4-phenylquinazoline (4d)

M.p. 228 °C, FT-IR (KBr, ν cm-1): 3057, 2925, 1606, 1580, 1537, 1518, 1481, 1445, 1388, 1349, 1080, 840, 711; ^1^H NMR (250 MHz, Chloroform-d) δ 8.87 (s, 2H), 8.36 (s, 2H), 8.25 – 8.04 (m, 2H), 7.87 (s, 3H), 7.65 (s, 3H); ^13^C NMR (63 MHz, DMSO-*d*_6_) δ 168.1, 158.2, 155.3, 150.2, 149.3, 143.5, 135.0, 131.0, 130.5, 130.0, 129.5, 128.8, 127.6, 126.4, 125.9, 124.1, 123.7, 122.5.


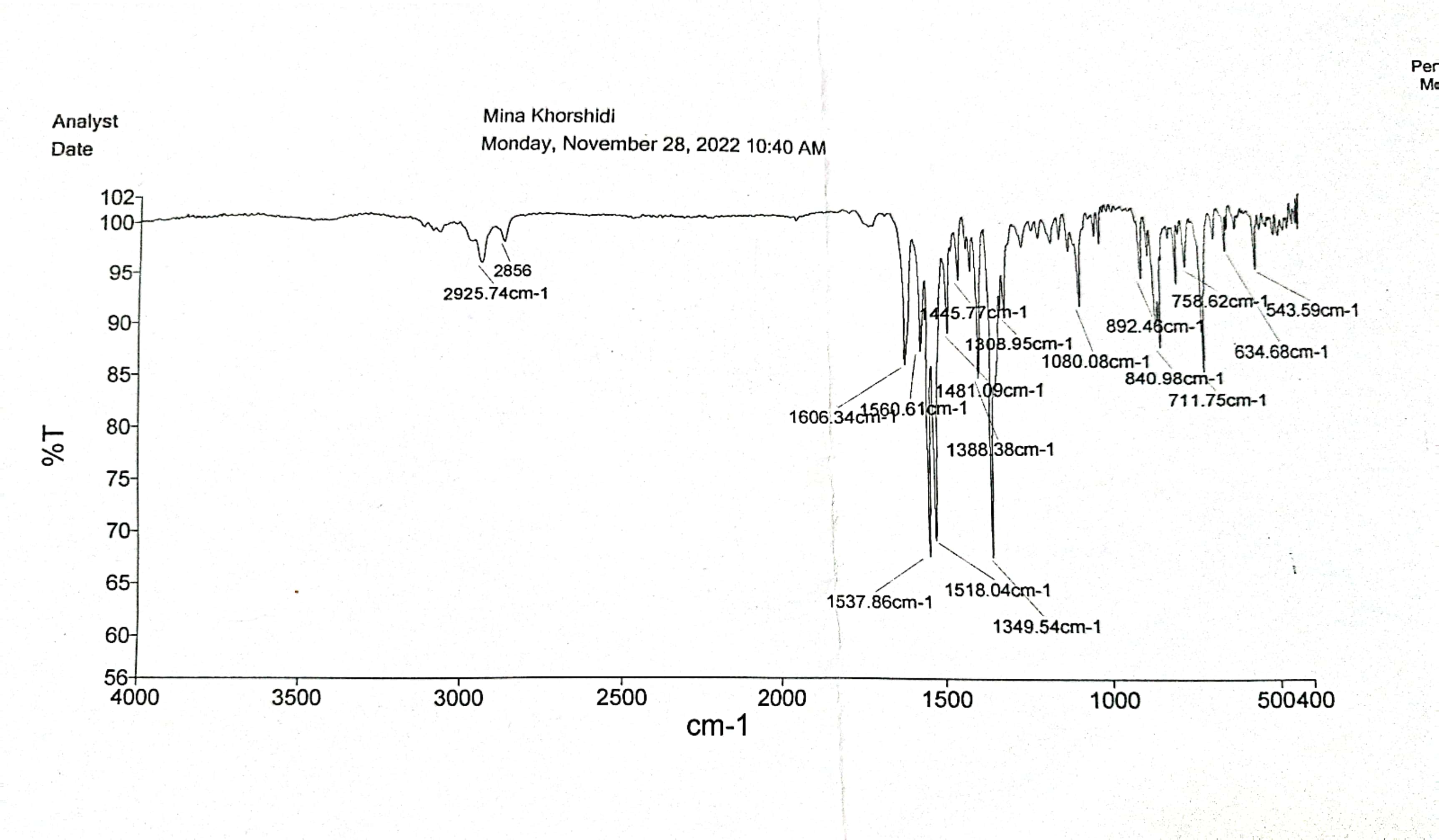


**Figure 11.** The FTIR spectrum of 6-chloro-2-(3-nitrophenyl)-4-phenylquinazoline (4d)


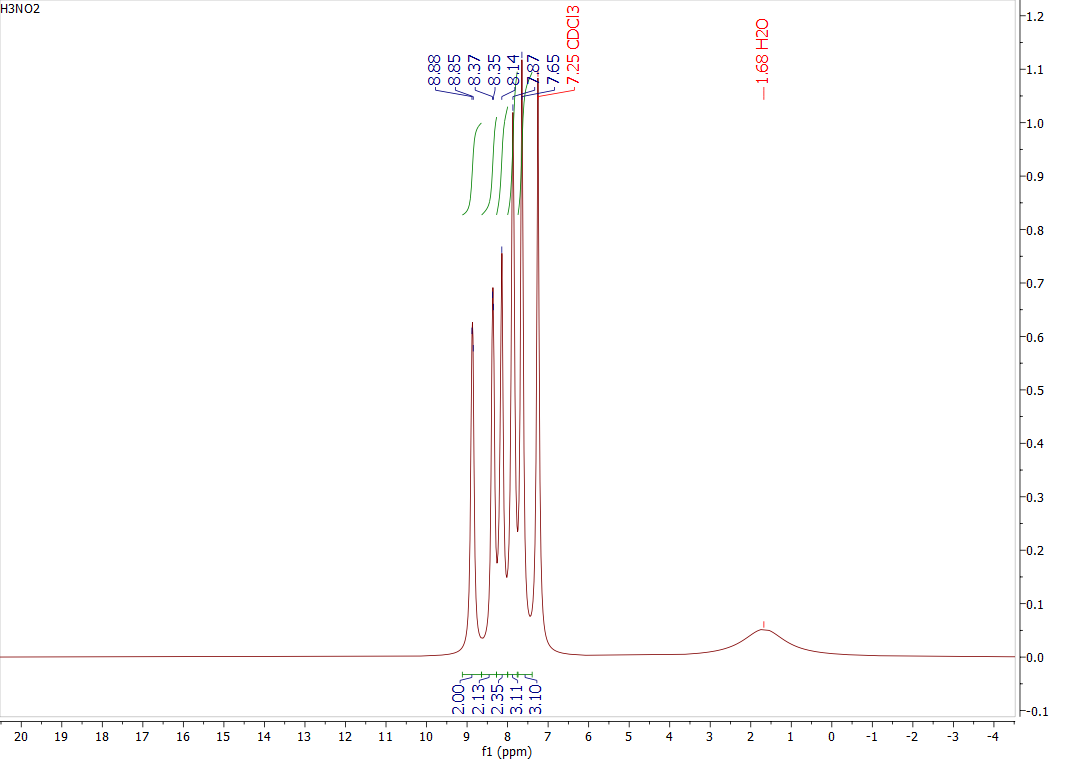


**Figure 12.** The ^1^H NMR (250 MHz) spectrum of *6-chloro-2-(3-nitrophenyl)-4-phenylquinazoline* in CDCl_3_ solvent (4d)


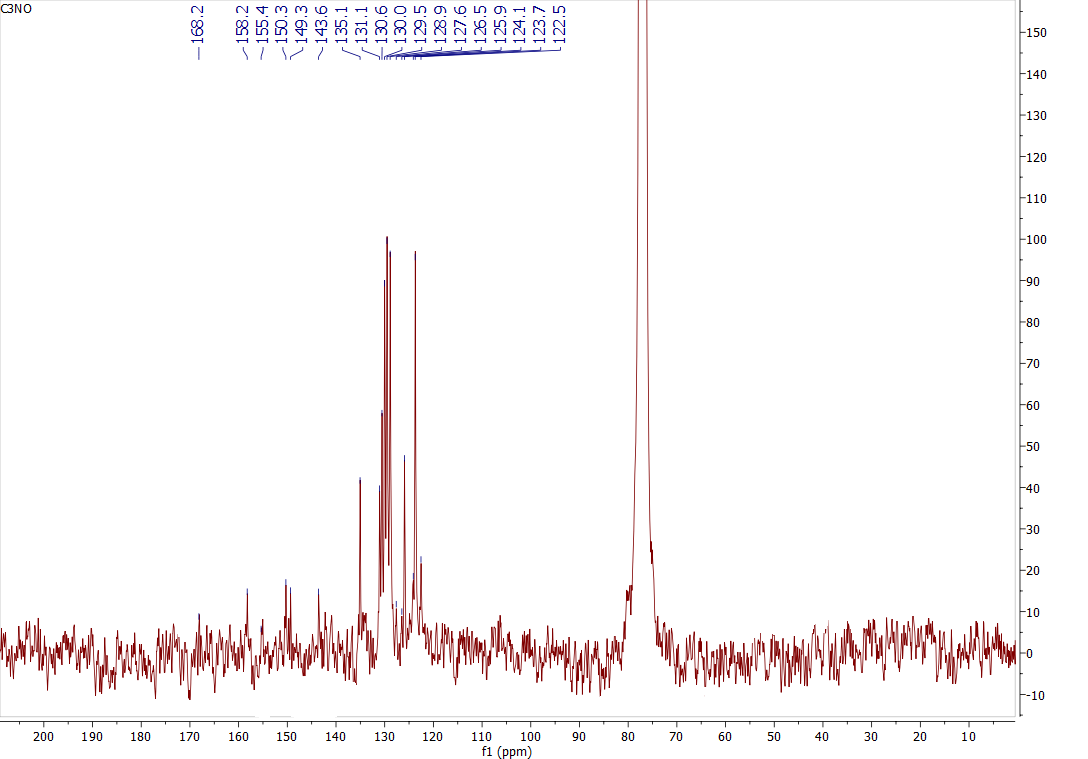


**Figure 13.** The CNMR (63 MHz) spectrum of 6-chloro-2-(3-nitrophenyl)-4-phenylquinazoline in CDCl_3_ solvent (4d)

6-chloro-2-(4-nitrophenyl)-4-phenylquinazoline (4e)

M.p. 218-220 °C, ^1^H NMR (250 MHz, Chloroform-d) δ 8.80 (d, J = 8.4 Hz, 2H), 8.41 (d, J = 8.4 Hz, 2H), 8.05 (s, 1H), 7.91 (d, J = 5.2 Hz, 2H), 7.68 (d, J = 5.3 Hz, 3H), 7.53 (s, 2H).


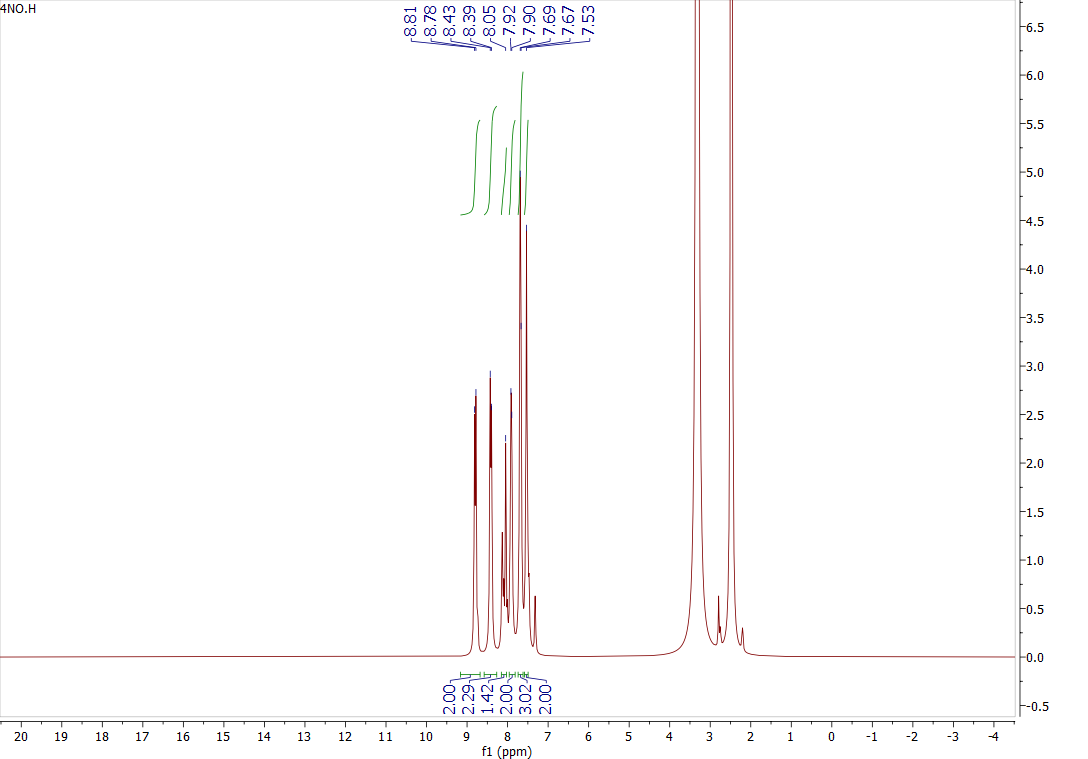


**Figure 14.** The ^1^H NMR (250 MHz) spectrum of *6-chloro-2-(4-nitrophenyl)-4-phenylquinazoline* in DMSO solvent (4e)

6-chloro-2-(4-fluorophenyl)-4-phenylquinazoline (4f)

M.p. 196 °C, FT-IR (KBr, ν cm-1): 3057, 1602, 1560, 1539, 1509, 1479, 1415, 1386, 1340, 1222, 1149, 890, 843; ^1^H NMR (250 MHz, DMSO-d) δ 8.80 – 8.37 (m, 2H), 8.03 (d, J = 15.7 Hz, 2H), 7.88 (s, 3H), 7.66 (s, 3H), 7.40 (s, 2H); ^13^C NMR (63 MHz, DMSO-*d*_6_) δ 161.60, 158.97, 150.20, 146.59, 140.36, 136.95, 135.51, 134.47, 132.43, 131.37, 131.01, 130.34, 129.82, 129.28, 125.91, 116.36.


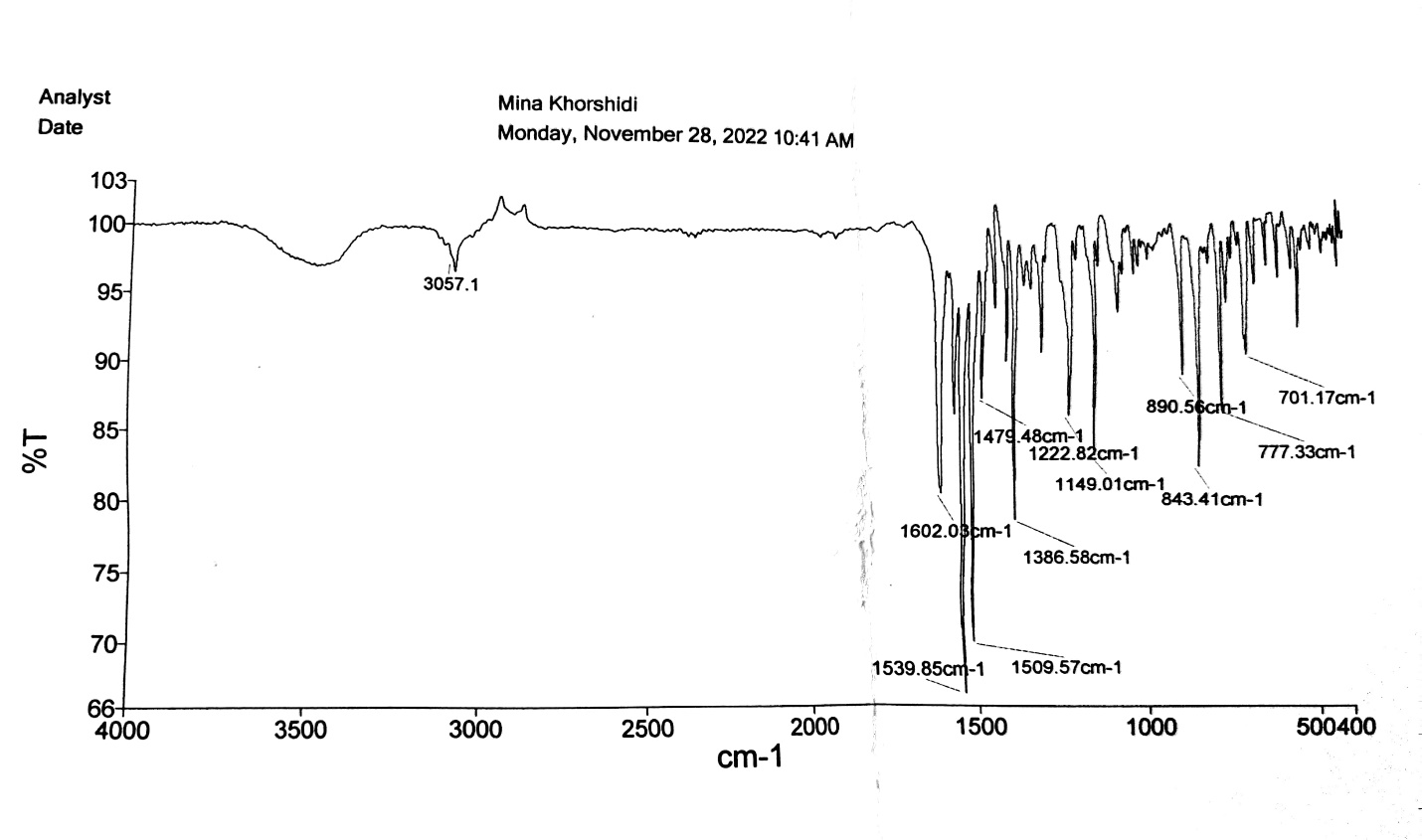


**Figure 15.** The FTIR spectrum of 6-chloro-2-(4-fluorophenyl)-4-phenylquinazoline (4f)


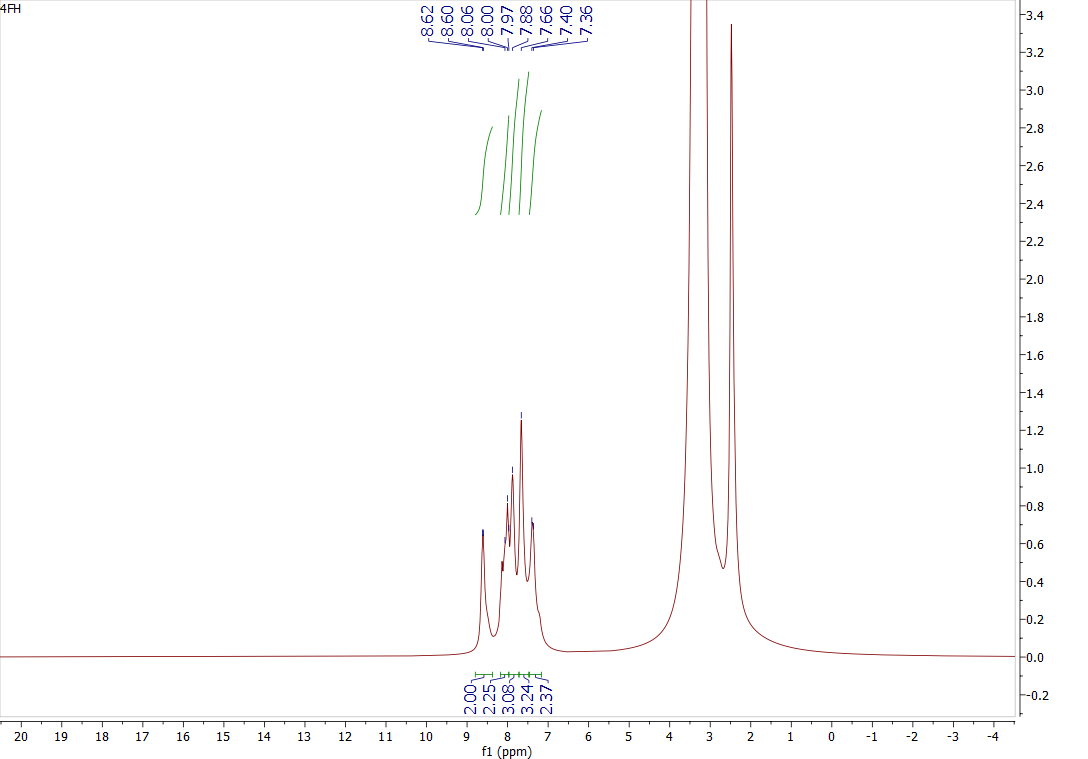


**Figure 16.** The ^1^H NMR (250 MHz) spectrum of *6-chloro-2-(4-fluorophenyl)-4-phenylquinazoline* in DMSO solvent (4f)


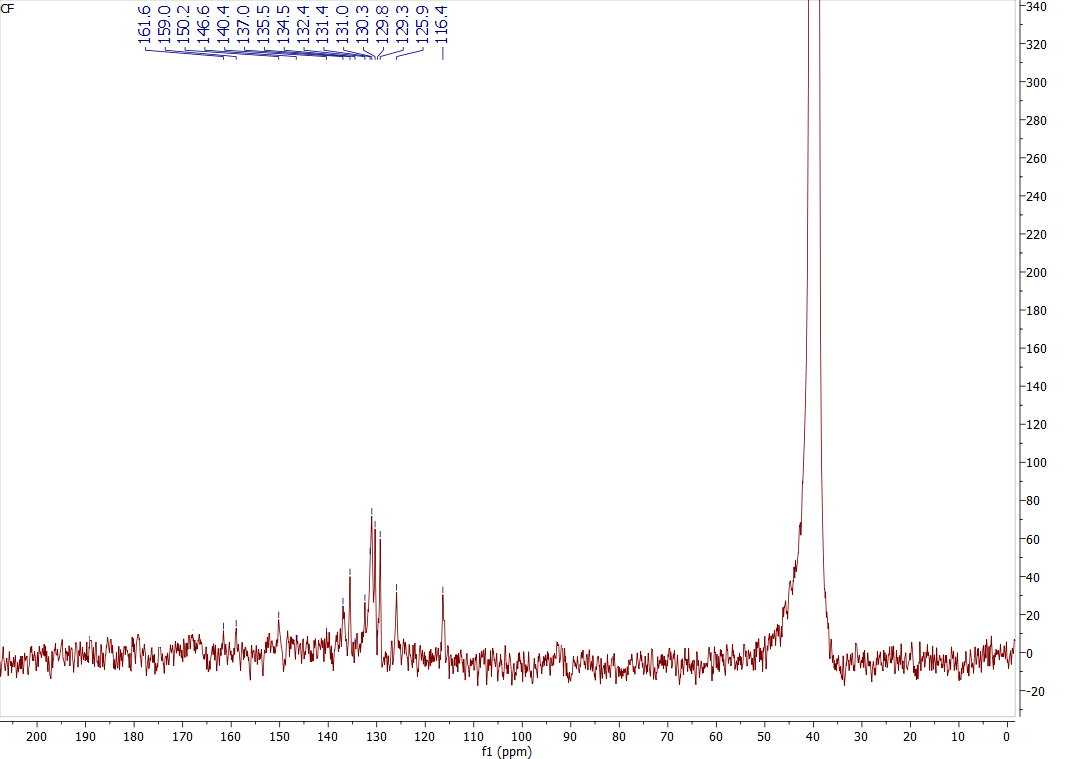


**Figure 17.** The CNMR (63 MHz) spectrum of 6-chloro-2-(4-fluorophenyl)-4-phenylquinazoline in DMSO solvent (4f)

6-chloro-2-(2,4-dichlorophenyl)-4-phenylquinazoline (4g)

M.p. 162-164 °C, ^1^H NMR (250 MHz, Chloroform-d) δ 8.55 – 8.05 (m, 2H), 7.88 (s, 4H), 7.60 (s, 4H), 7.40 (s, 1H); ^13^C NMR (63 MHz, DMSO-*d*_6_) δ 160.4, 154.1, 150.0, 147.5, 136.5, 135.8, 134.9, 132.8, 131.7, 130.8, 130.4, 130.0, 128.8, 127.2, 125.8.


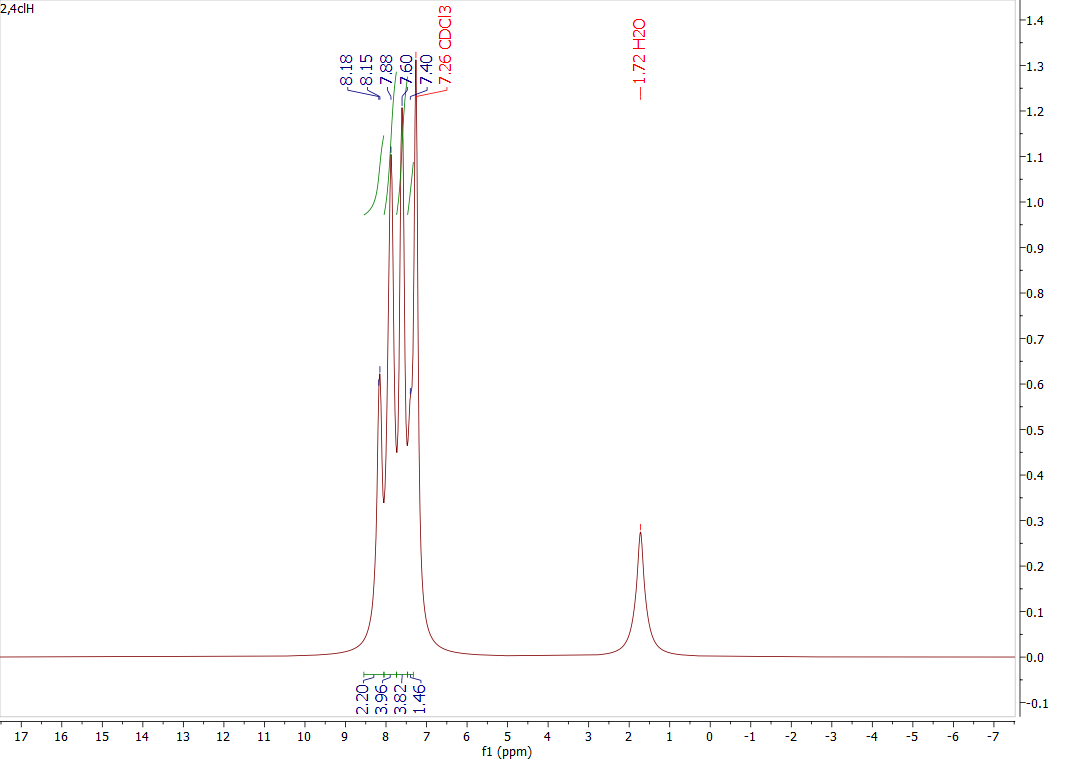


**Figure 18.** The ^1^H NMR (250 MHz) spectrum of *6-chloro-2-(2,4-dichlorophenyl)-4-phenylquinazoline* in CDCl_3_ solvent (4g)


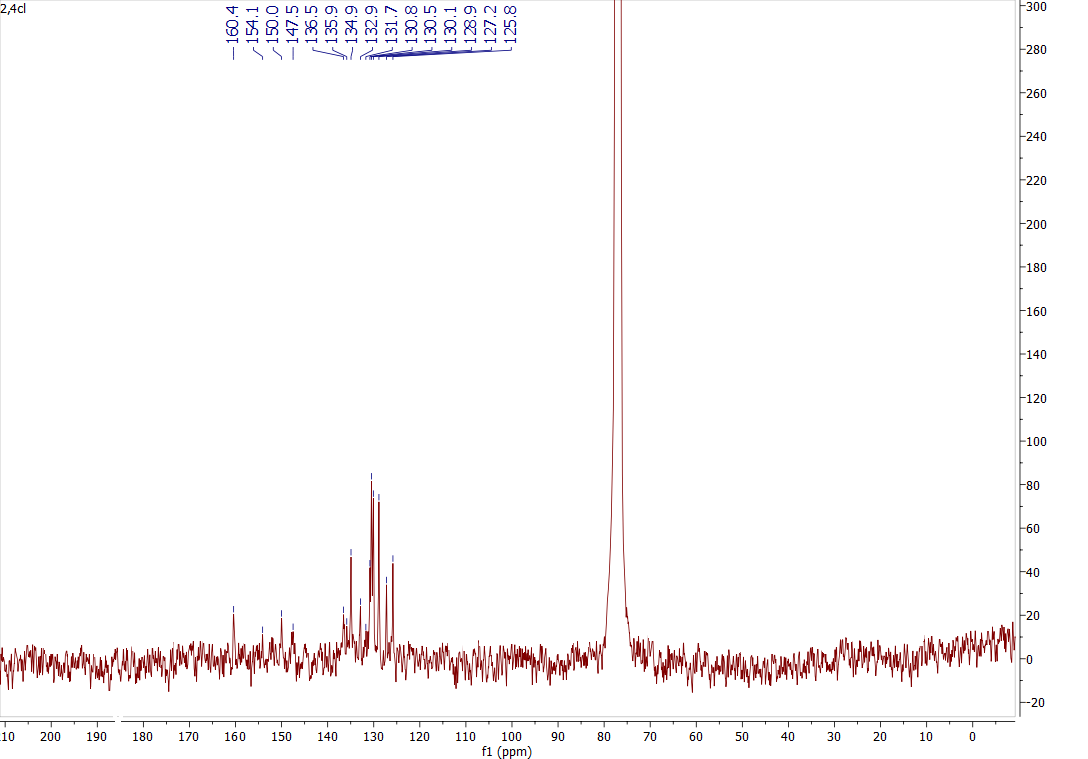


**Figure 19.** The CNMR (63 MHz) spectrum of *6-chloro-2-(2,4-dichlorophenyl)-4-phenylquinazoline* in CDCl_3_ solvent (4g)

6-chloro-2-(furan-2-yl)-4-phenylquinazoline (4h)

M.p. 194-196 °C, FT-IR (KBr, ν cm-1): 3055, 1609, 1557, 1539, 1471, 1402, 1378, 1240, 1157, 887, 751; ^1^H NMR (250 MHz, Chloroform-d) δ 8.07 (s, 2H), 7.81 (s, 3H), 7.60 (s, 5H), 6.61 (s, 1H); ^13^C NMR (63 MHz, DMSO-*d*_6_) δ 168.0, 153.7, 152.4, 150.1, 145.8, 134.8, 130.5, 130.3, 129.9, 128.7, 128.3, 125.9, 122.1, 114.6, 112.2.


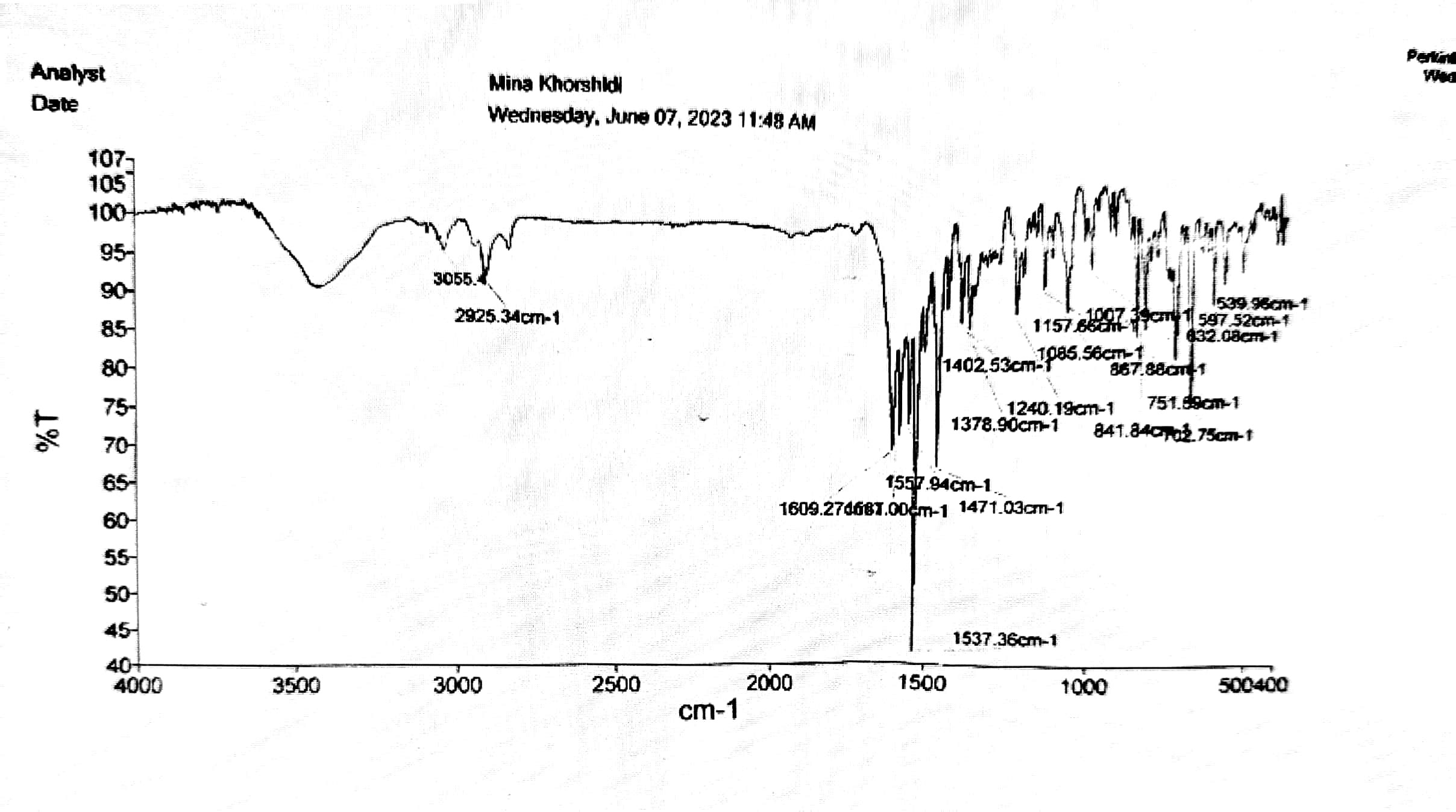


**Figure 20.** The FTIR spectrum of *6-chloro-2-(furan-2-yl)-4-phenylquinazoline* (4h)


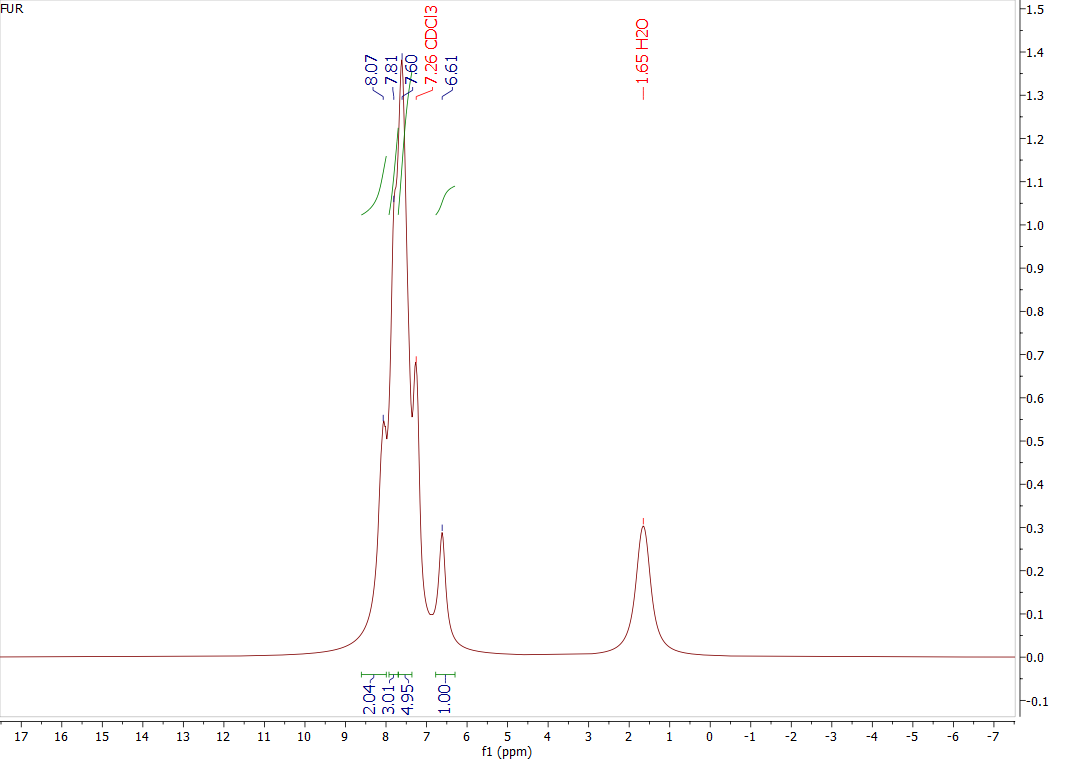


**Figure 21.** The ^1^H NMR (250 MHz) spectrum of *6-chloro-2-(furan-2-yl)-4-phenylquinazoline* in CDCl_3_ solvent (4h)


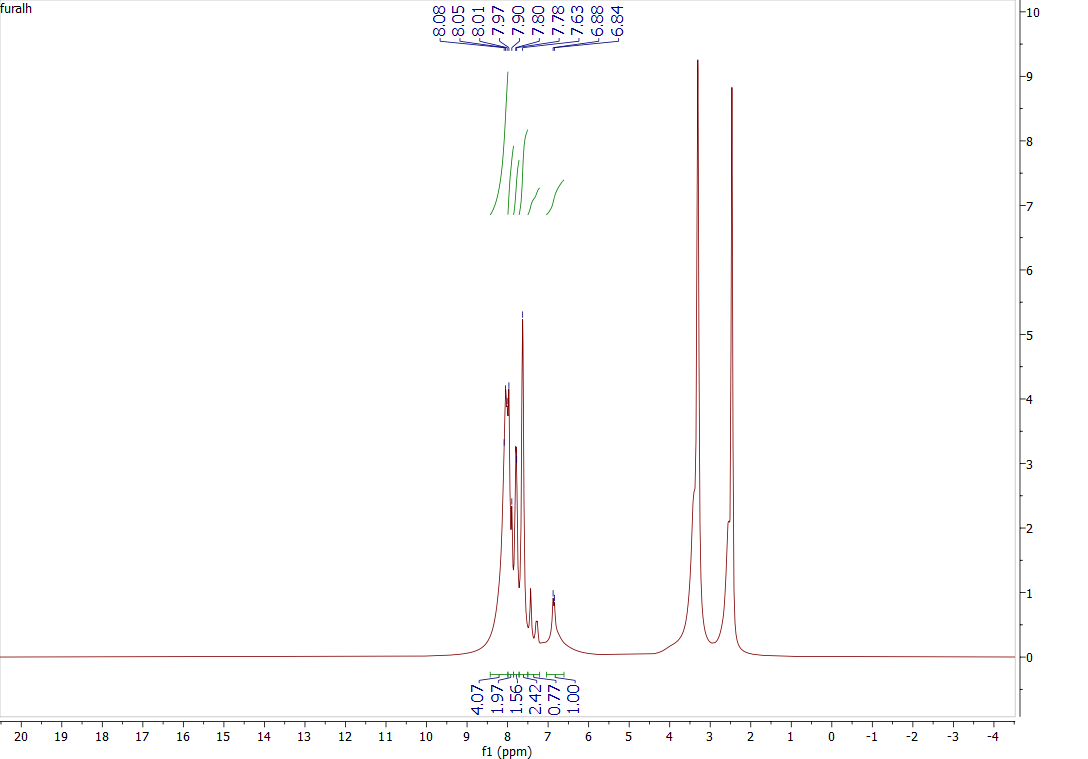


**Figure 22.** The ^1^H NMR (250 MHz) spectrum of *6-chloro-2-(furan-2-yl)-4-phenylquinazoline* in DMSO solvent (4h)


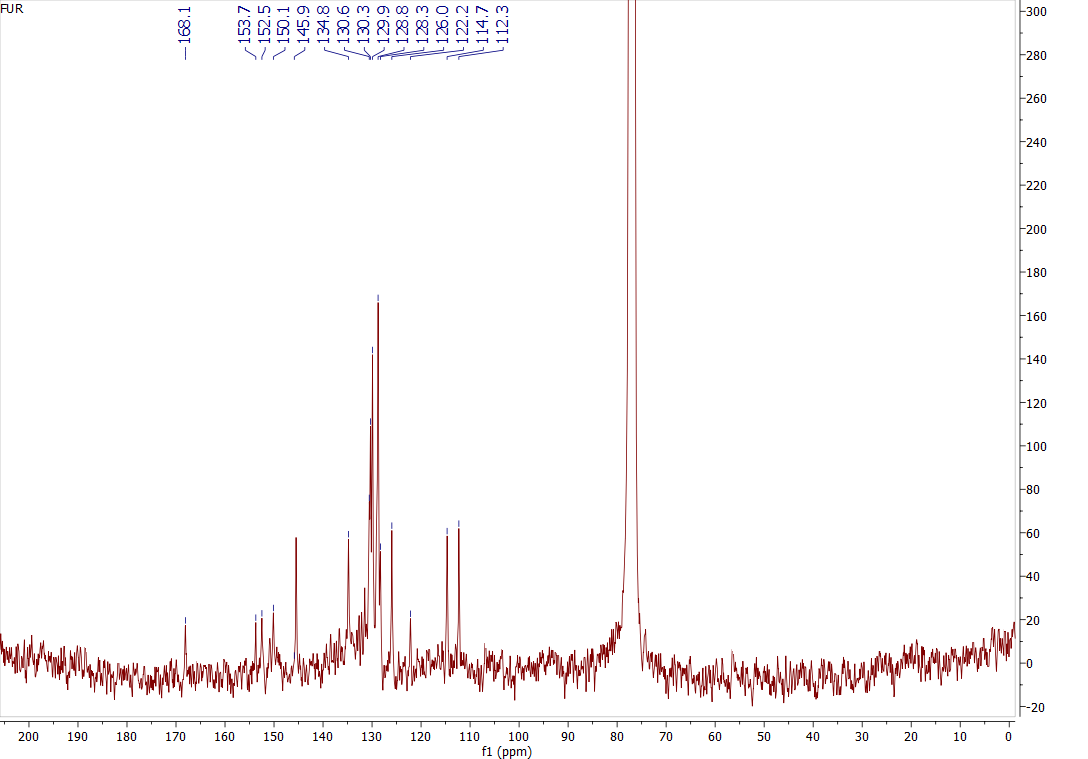


**Figure 23.** The CNMR (63 MHz) spectrum of 6-chloro-2-(furan-2-yl)-4-phenylquinazoline in CDCl_3_ solvent (4h)

6-chloro-4-phenyl-2-(thiophen-2-yl)quinazoline (4i)

M.p. 219-220 °C, ^1^H NMR (250 MHz, Chloroform-d) δ δ 8.18 (s, 2H), 8.06 (d, J = 14.9 Hz, 2H), 7.82 (s, 3H), 7.56 (d, J = 24.8 Hz, 3H), 7.17 (s, 1H); ^13^C NMR (63 MHz, DMSO-*d*_6_) δ 157.4, 155.0, 150.4, 144.4, 142.5, 137.0, 134.6, 132.2, 130.3, 130.1, 130.0, 129.6, 128.7, 128.2, 125.9.


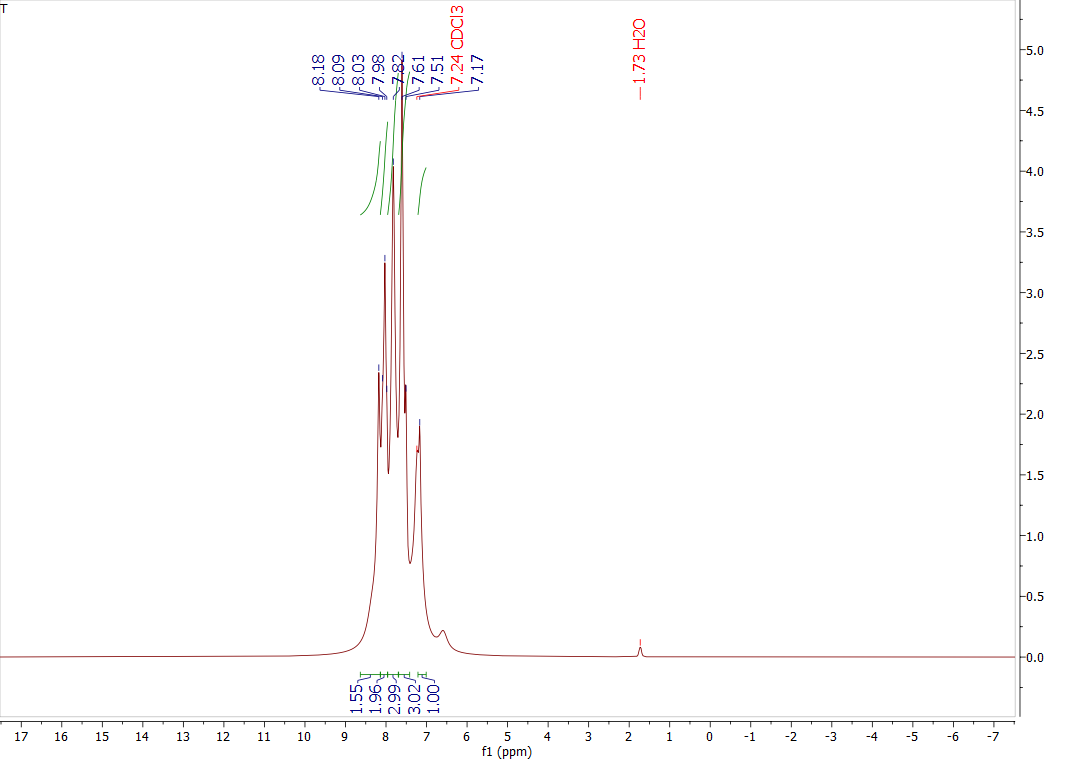


**Figure 24.** The ^1^H NMR (250 MHz) spectrum of *6-chloro-4-phenyl-2-(thiophen-2-yl)quinazoline* in CDCl_3_ solvent (4i)


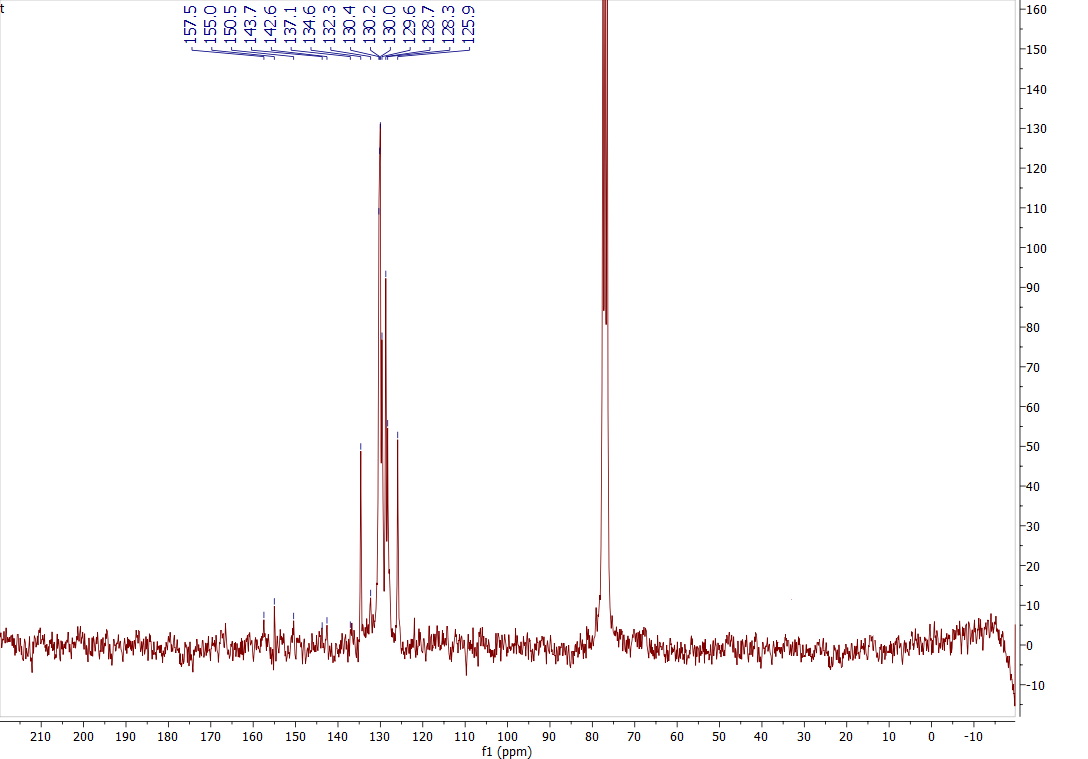


**Figure 25.** The CNMR (63 MHz) spectrum of 6-chloro-4-phenyl-2-(thiophen-2-yl)quinazoline in CDCl_3_ solvent (4i)

6-chloro-2-(4-methoxyphenyl)-4-phenylquinazoline (4j)

M.p. 194-196 °C, FT-IR (KBr, ν cm-1): 3051, 1655, 1608, 1534, 1512, 1415, 1337, 1250, 1162, 1029, 833, 702; ^1^H NMR (250 MHz, Chloroform-d) δ 8.63 (d, J = 8.5 Hz, 2H), 8.03 (d, J = 8.2 Hz, 2H), 7.81 (dd, J = 20.3, 7.2 Hz, 3H), 7.61 (s, 3H), 7.03 (d, J = 8.5 Hz, 2H), 3.89 (s, 3H).^13^C NMR (63 MHz, CDCl3) δ 167.3, 161.9, 160.2, 150.5, 137.2, 134.3, 132.0, 131.2, 130.6, 130.3, 130.0, 128.6, 125.7, 121.8, 113.9, 55.4.


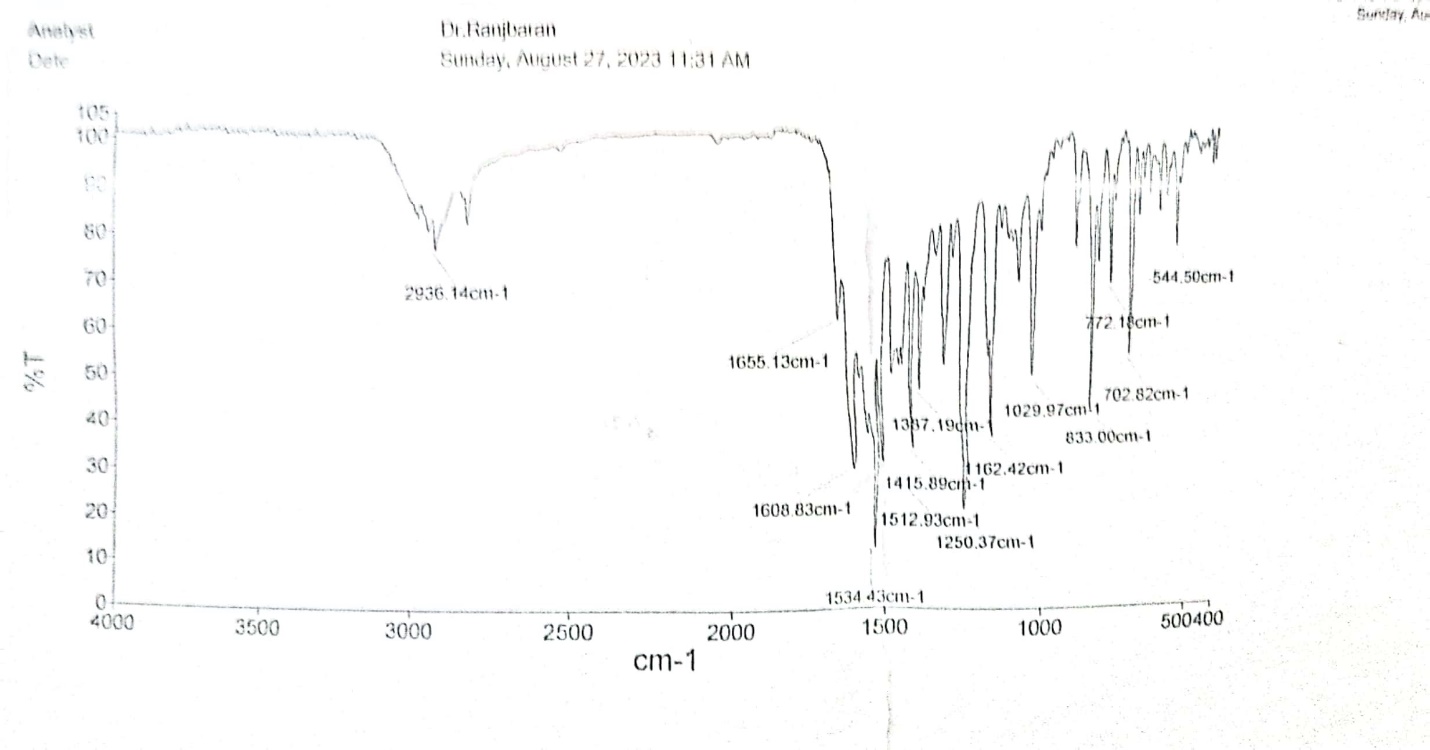


**Figure 26.** The FTIR spectrum of *6-chloro-2-(4-methoxyphenyl)-4-phenylquinazoline* (4j)


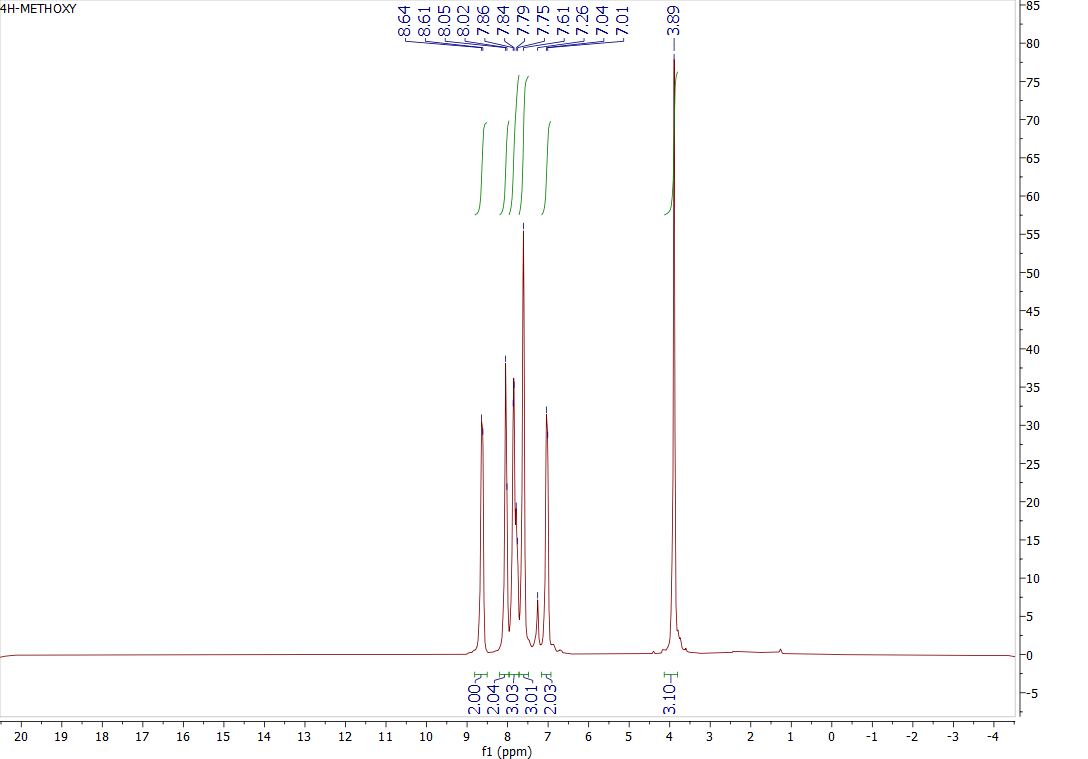


**Figure 27.** The ^1^H NMR (250 MHz) spectrum of *6-chloro-2-(4-methoxyphenyl)-4-phenylquinazoline* in CDCl_3_ solvent (4j)


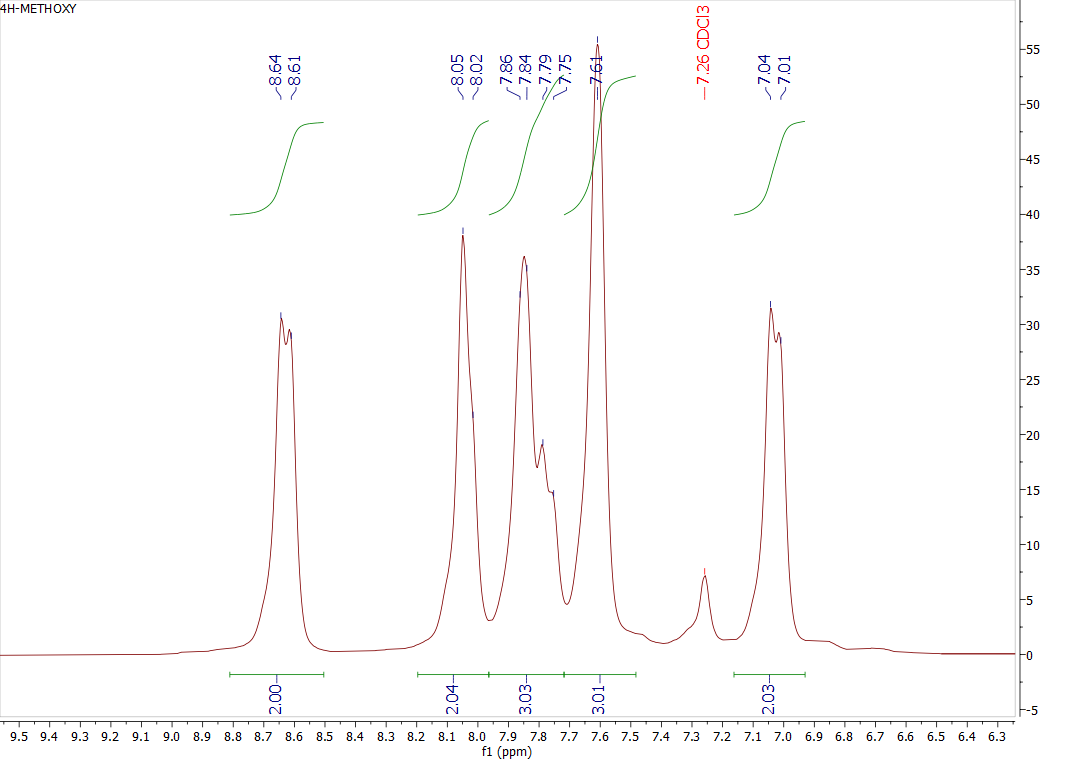


**Figure 28.** Magnification of the ^1^H NMR (250 MHz) spectrum of *6-chloro-2-(4-methoxyphenyl)-4-phenylquinazoline* in CDCl_3_ solvent (4j)


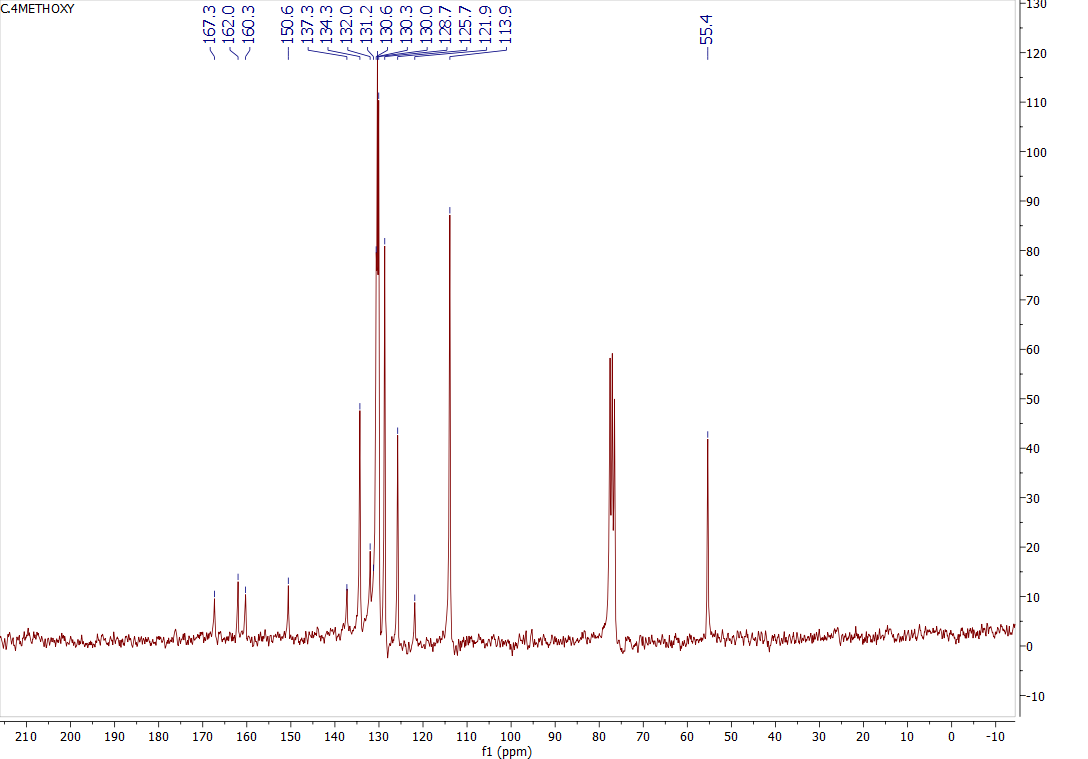


**Figure 29.** The CNMR (63 MHz) spectrum of *6-chloro-2-(4-methoxyphenyl)-4-phenylquinazoline* in CDCl_3_ solvent (4j)

4-(6-chloro-4-phenylquinazolin-2-yl)phenol (4k)

M.p. 194-196 °C, FT-IR (KBr, ν cm-1): 3480, 3076, 1613, 1591, 1562, 1531, 1479, 1416, 1390, 1278, 1163, 829, 699; ^1^H NMR (250 MHz, DMSO-d6) δ 9.77 (s, 1H), 8.41 (d, J = 7.7 Hz, 2H), 8.06 (d, J = 8.1 Hz, 1H), 7.99 (s, 1H), 7.93 (d, J = 5.2 Hz, 1H), 7.84 (s, 2H), 7.65 (s, 3H), 6.91 (d, J = 7.6 Hz, 2H).^13^C NMR (63 MHz, DMSO) δ 160.9, 160.1, 150.4, 146.3, 142.6, 137.0, 135.1, 131.5, 131.1, 130.6, 130.3, 129.2, 125.8, 121.7, 116.0.


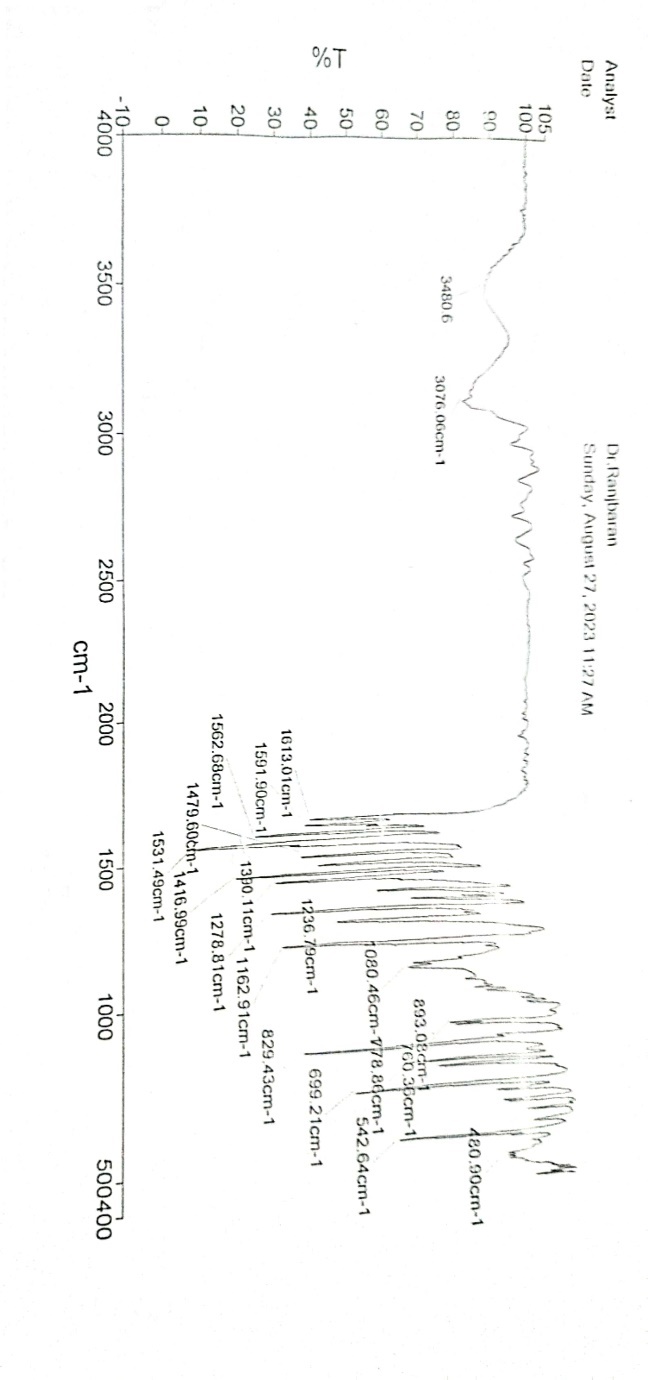


**Figure 30.** The FTIR spectrum of *4-(6-chloro-4-phenylquinazolin-2-yl)phenol* (4k)


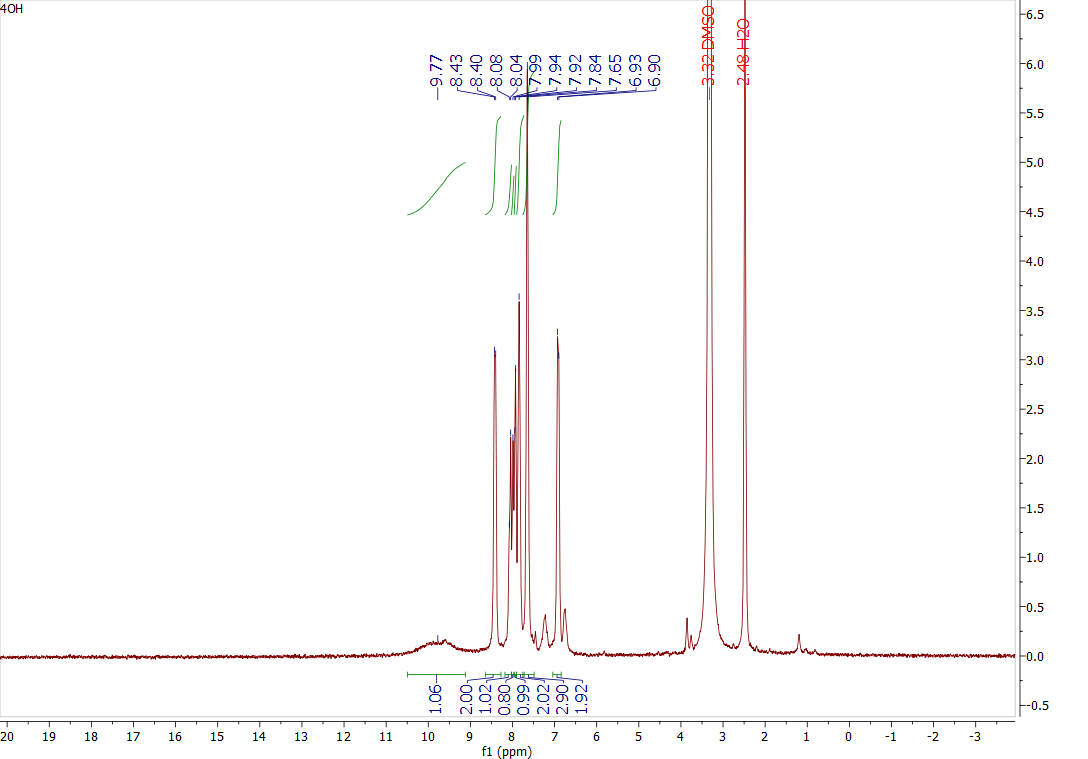


**Figure 31.** The ^1^H NMR (250 MHz) spectrum of *4-(6-chloro-4-phenylquinazolin-2-yl)phenol* in DMSO solvent (4k)


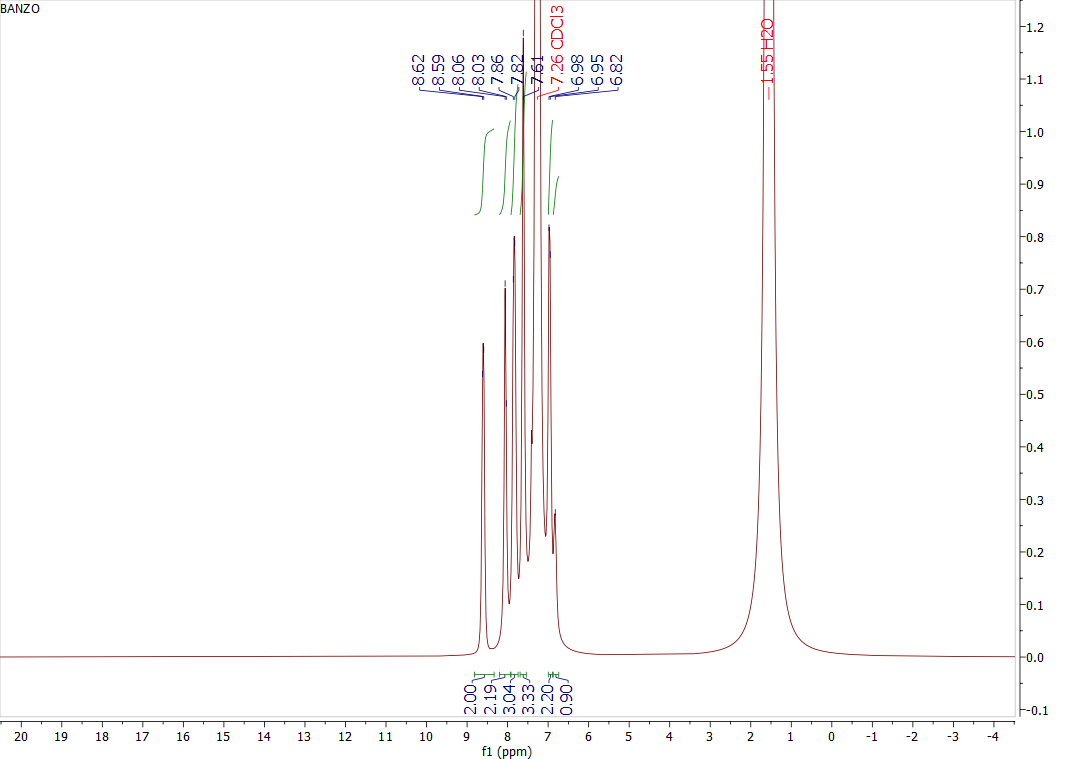


**Figure 32.** The ^1^H NMR (250 MHz) spectrum of *4-(6-chloro-4-phenylquinazolin-2-yl)phenol* in CDCl_3_ solvent (4k)


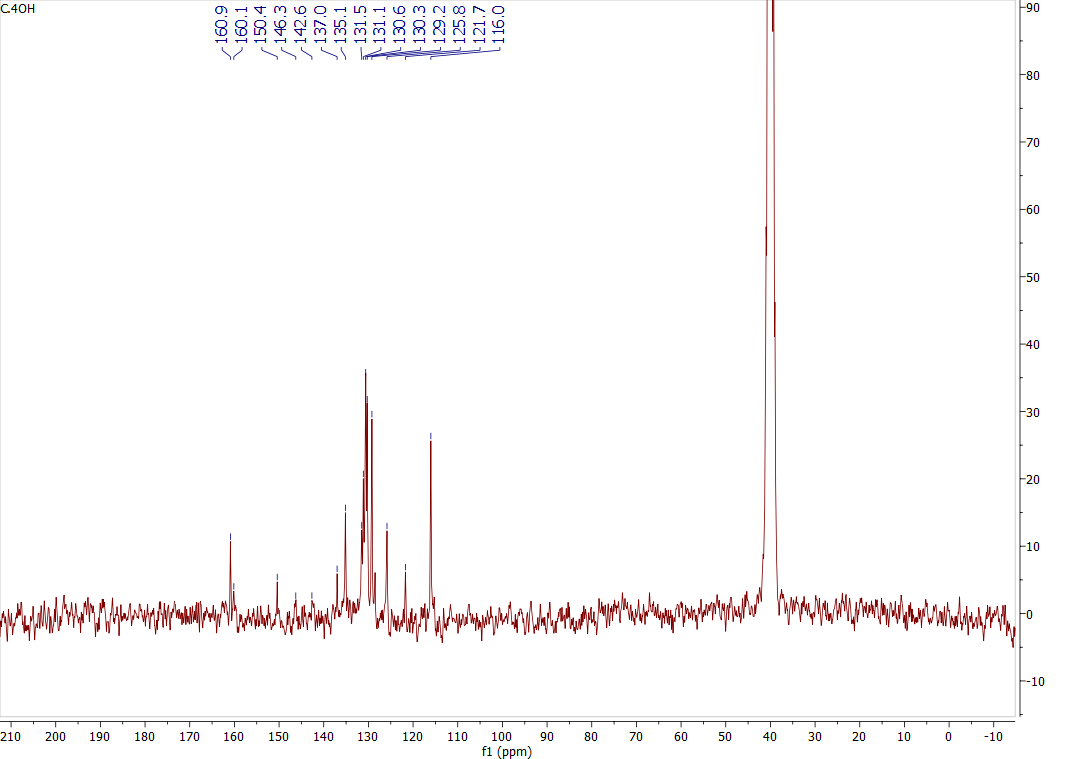


**Figure 33.** The CNMR (63 MHz) spectrum of *4-(6-chloro-4-phenylquinazolin-2-yl)phenol* in DMSO solvent (4k)

2-(6-chloro-4-phenylquinazolin-2-yl)benzoic acid (4l)

M.p. 194-196 °C, FT-IR (KBr, ν cm-1): 3437, 3061, 2930, 1736, 1686, 1561, 1541, 1474, 1384, 1240, 1077, 840, 702; ^1^H NMR (250 MHz, Chloroform-d) δ 8.68 (d, J = 9.2 Hz, 2H), 7.96 (s, 3H), 7.70 (s, 3H), 7.59 (s, 2H), 7.02 (s, 2H), 6.06 (s, 1H).; ^13^CNMR (63 MHz, DMSO) δ 166.5, 162.1, 152.1, 150.1, 143.4, 137.5, 135.1, 134.7, 134.4, 133.1, 132.5, 131.4, 131.1, 130.7, 129.9, 129.6, 129.2, 125.9, 125.7.


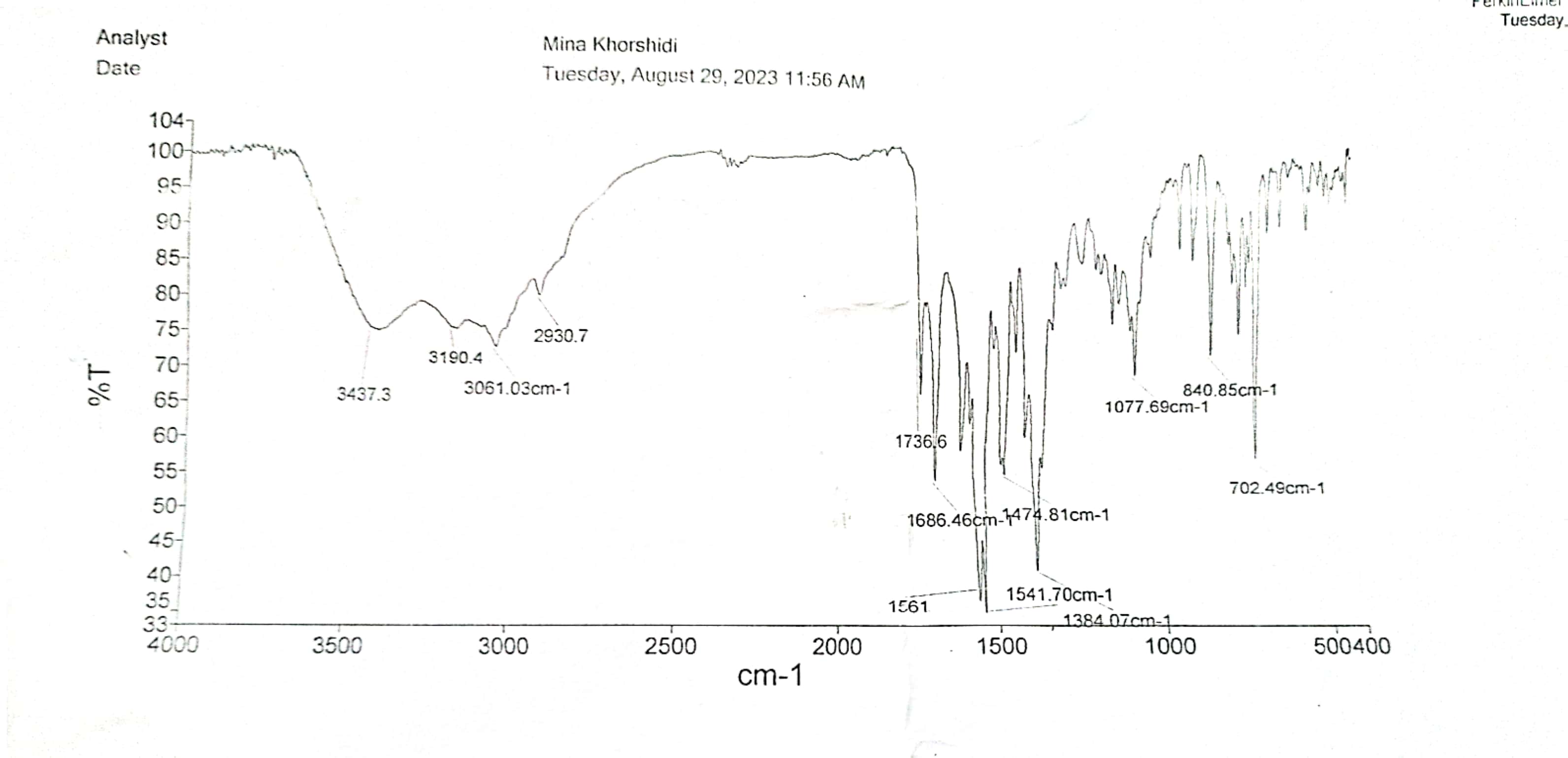


**Figure 34.** The FTIR spectrum of *2-(6-chloro-4-phenylquinazolin-2-yl)benzoic acid* (4l)


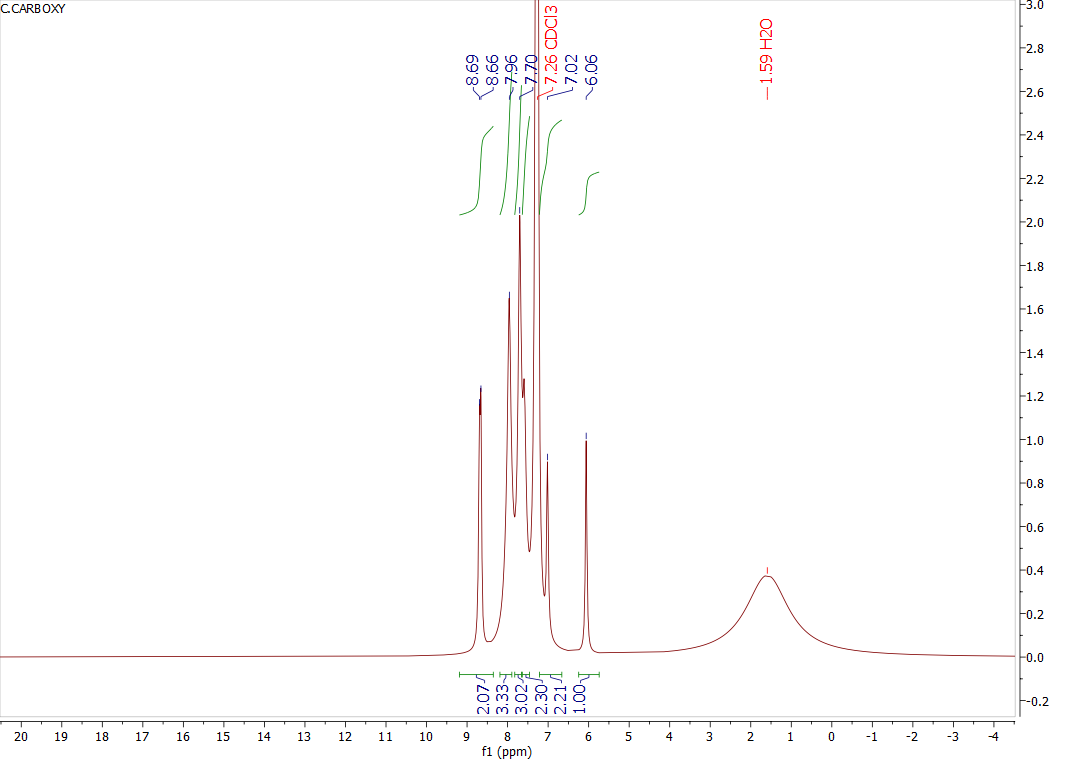


**Figure 35.** The ^1^H NMR (250 MHz) spectrum of *2-(6-chloro-4-phenylquinazolin-2-yl)benzoic acid*  in CDCl_3_ solvent (4l)


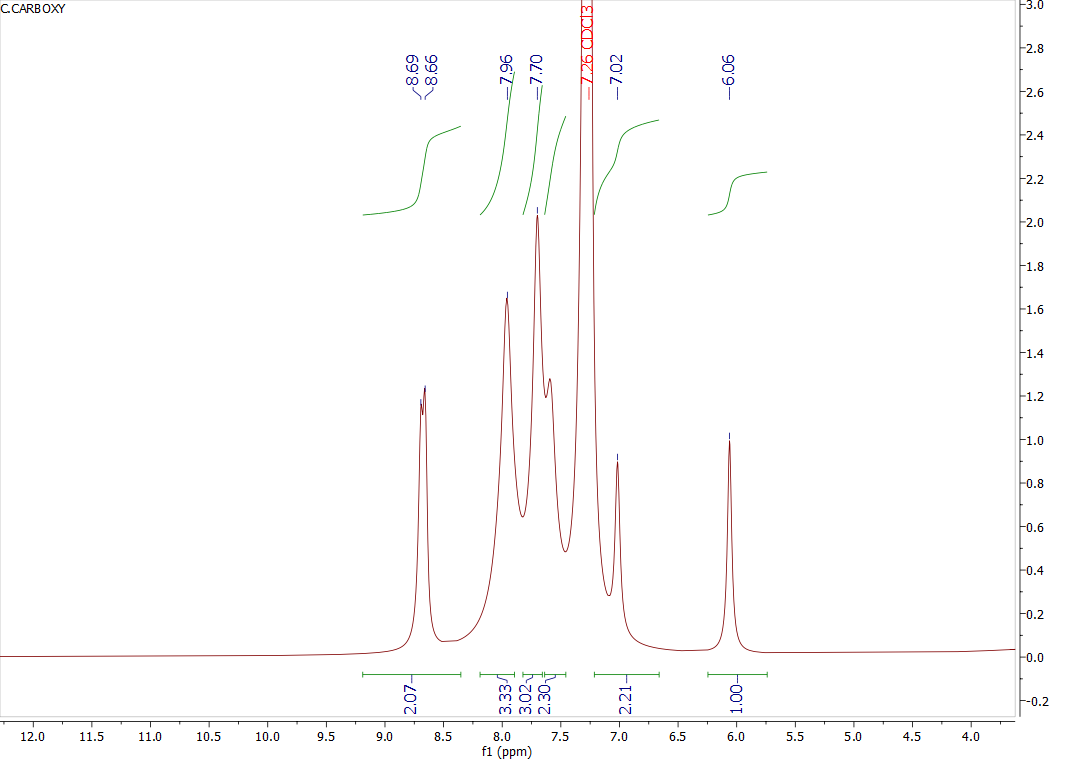


**Figure 36.** Magnification of the ^1^H NMR (250 MHz) spectrum of *2-(6-chloro-4-phenylquinazolin-2-yl)benzoic acid* in CDCl_3_ solvent (4l)


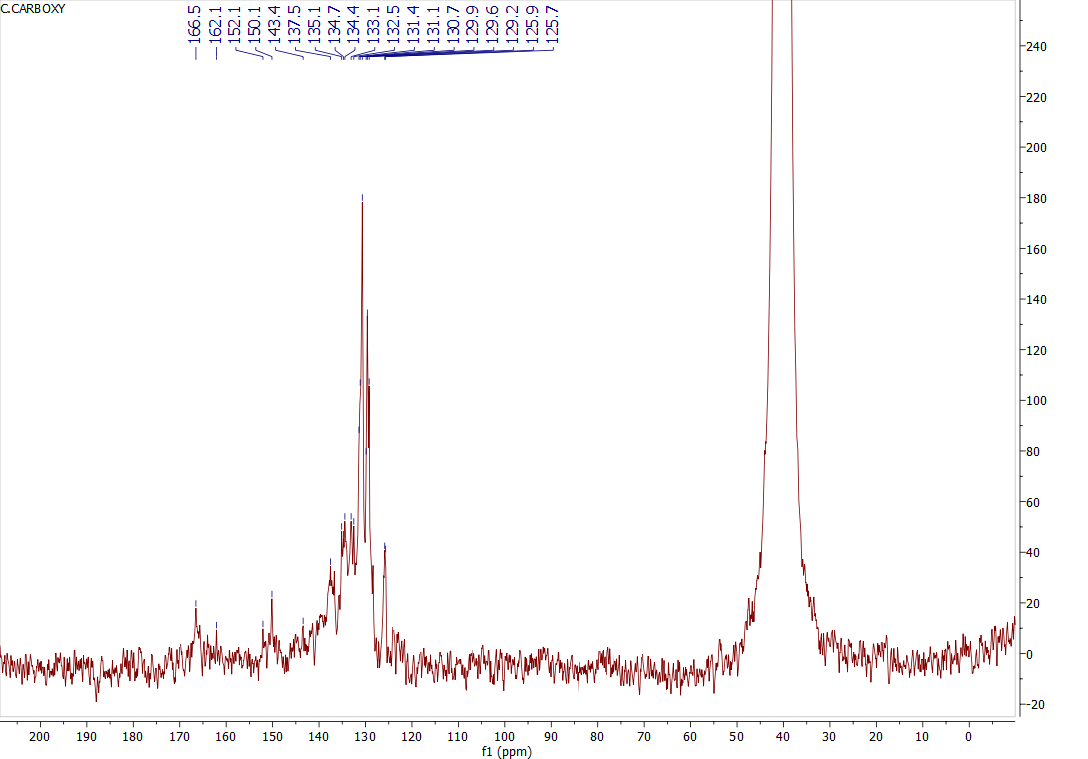


**Figure 37.** The CNMR (63 MHz) spectrum of 2-(6-chloro-4-phenylquinazolin-2-yl)benzoic acid in DMSO solvent (4l)
